# An Integrated Pharmacology-Based Analysis for Antidepressant Mechanism of Chinese Herbal Formula Xiao-Yao-San

**DOI:** 10.3389/fphar.2020.00284

**Published:** 2020-03-18

**Authors:** Naijun Yuan, Lian Gong, Kairui Tang, Liangliang He, Wenzhi Hao, Xiaojuan Li, Qingyu Ma, Jiaxu Chen

**Affiliations:** ^1^Formula-Pattern Research Center, School of Traditional Chinese Medicine, Jinan University, Guangzhou, China; ^2^College of Pharmacy, Jinan University, Guangzhou, China

**Keywords:** network pharmacology, Xiao-Yao-San, depression, Chinese herb formula, integration analysis methods

## Abstract

Clinical studies and basic science experiments have widely demonstrated the antidepressant and anxiolytic effects of the herbal formula Xiao-Yao-San (XYS). However, the system mechanism of these effects has not been fully characterized. The present study conducted a comprehensive network pharmacological analysis of XYS and sorted all pharmacologically active components (149) through the TCMSP webserver. Then, all potential molecular targets (449) were predicted, of which there were 99 genes clearly related to depression. To further investigate the mechanism of antidepressant effects of XYS, a compound-depression targets (C-DTs) network was constructed, and Gene Ontology (GO) functional and KEGG pathway enrichment analyses were performed for the 99 targets. Enrichment results revealed that XYS could regulate multiple aspects of depression through these targets, related to metabolism, neuroendocrine function, and neuroimmunity. Prediction and analysis of protein–protein interactions resulted in selection of three hub genes (AKT1, TP53, and VEGFA). In addition, a total of seven ingredients from XYS could act on these hub genes and they were identified through ultra-high-performance liquid chromatography-quadrupole time-of-flight mass spectrometry (UPLC-Q/TOF-MS), including paeoniflorin, quercetin, luteolin, acacetin, aloe-emodin, Glyasperin C, kaempferol. Hereafter, we investigated the effects of paeoniflorin and its predicted target, the results suggest that it can reverse the neurotoxicity produced by CORT and could be a neuroprotective effect by promoting the phosphorylation of Akt. Overall, our research revealed the complicated antidepressant mechanism of XYS, and also provided a rational strategy for revealing the complex composition and function of Chinese herbal formula.

## Introduction

Depression is a psychological disorder with widespread prevalence, affecting more than 350 million individuals worldwide, and the prevalence is gradually increasing (Kuo et al., 2015). Because of high morbidity and mortality, depression has received extensive attention. Currently, the main treatment strategy for depression is pharmacotherapy, but antidepressants typically do not completely alleviate depressive symptoms and may lead to drug addiction and adverse side effects for patients (Tom et al., 2014; Solem et al., 2017).

Development of medicines that have antidepressant effects and produce less side effects than current antidepressants is a priority. Therefore, complementary and alternative medicine (CAM) and traditional Chinese medicine (TCM) have received attention as potential strategies for treatment and prevention of depression. Xiao-Yao-San (XYS) has been used in TCM clinics for more than a century to treat various diseases with characteristic features of liver stagnation and spleen deficiency syndrome (LSSDS) (Meng et al., 2013). There are eight Chinese herbs in XYS: *Angelica sinensis* (Oliver) Diels (AS, family: Apiaceae); *Paeonia lactiflora* Pallas (PN, family: Paeoniaceae); *Bupleurum Chinense* De Candolle (BR, family: Apiaceae); *Atractylodes macrocephala* Koidzumi (AMR, family: Compositae); *Poria cocos* (Schweinitz) Wolf (PR, family: Polyporaceae); *Mentha canadensis* Linnaeus (MH; family: Lamiaceae); *Glycyrrhiza uralensis* Fischer (GRH; family: Leguminosea); *Zingiber officinale* Roscoe (ZRR, family: Zingiberaceae).

XYS has been widely prescribed as a safe and effective treatment or adjuvant therapy for depression, because psychological stress syndromes primarily are classified as LSSDS in TCM theory. Clinical studies and basic science experiments have demonstrated the antidepressant and anxiolytic effects of XYS (Dai et al., 2010; Chen and Hou, 2017), but the associated mechanisms have not been characterized.

Systems pharmacology is an emerging field that integrates bioinformatics and experimental methods to advance drug discovery and provides a method for clarification of mechanisms of action of Chinese herbs (Berger and Iyengar, 2009). A drug (active compounds)-targets-diseases (clinical symptoms) network can be constructed and analyzed using a holistic view though this method (Kim et al., 2019; Zhou et al., 2019). It can determine the enrichment of targets, and elucidate complex effects and pharmacological mechanisms of Chinese herbs (Kloft et al., 2016). The present study aimed to identify the bioactive components and mechanisms of action of the TCM formula XYS in the treatment of depression using system network pharmacological analysis.

## Materials and Methods

### Data Preparation

#### Construction of XYS Chemical Constituent Databases

All chemical constituents of each of the herbs in XYS were obtained from the TCMSP data server (Traditional Chinese Medicine Systems Pharmacology Database and Analysis Platform, http://lsp.nwu.edu.cn/tcmsp.php), which is a unique systems-level pharmacology server for TCM (Ru et al., 2014). TCMSP can also provide pharmacology-related properties and predict targets and related diseases for each active ingredient based on critically evaluated pharmacological and clinical knowledge. This ground-breaking data server highlights the role that systems pharmacology can play in the traditional Chinese medicine discipline. Moreover, ADME properties data were also derived from TCMSP, comprising molecular weight (MW), octanol-water coefficient (ALogP), number of possible hydrogen-bond donors (nHdon), and number of hydrogen-bond acceptors (nHacc).

#### Evaluation the Drug-Likeness

Potential drugs in XYS were mainly identified by integrating oral bioavailability (OB) and drug-likeness (DL) properties. Comprehensive analysis of bioavailability and structural descriptors for predicting OB values for each compound were previously determined *in-silico* (Xue et al., 2012). [OB%]. Data server-dependent DL models were used to determine solubility and chemical stability based on the Tanimoto coefficient, using the following formula: T (A, B) = (A×B)/(|A|^2^ + |B|^2^ − A×B) (Tao et al., 2013). Then, according to recommendations from TCMSP, we selected screening criteria of OB ≥ 30% and DL ≥ 0.18 for determination of possible bioactive components.

#### Known Antidepressant Drug/Drug-Like Compounds Database Construction

##### Known antidepressant drug/drug-like compounds from the cMap database

Gene profiling of depression was performed using GSE12654 from GEO database, which contains frozen brain tissues from 11 depressed individuals and brain tissues from 15 non-depressed individuals (Iwamoto et al., 2004). Differentially expressed genes (DEGs) with the criteria |logFC|>1.2 and *P* < 0.05 were determined by “limma” package in R studio software. Moreover, the connectivity map (cMap) database is a collection of genome-wide transcriptional expression data from cultured human cells treated with bioactive small molecules that uses simple pattern-matching algorithms allowing for elucidation of functional connections between drugs, genes, and diseases through transitory common gene expression changes (Fang et al., 2003). After determining dysregulated genes, we converted the associated gene symbols to Affymetrix probe IDs, as the cMap database is based on these identifiers. *P* < 0.05 was used as the threshold for statistical significance.

##### Known antidepressant drug/drug-like compounds from DrugBank database

DrugBank (http://www.drugbank.ca/) is a unique online database used as a bioinformatics and cheminformatics resource that compiles extensive drug-related information (Wishart et al., 2017). Therefore, DrugBank was also searched for antidepressant drugs or drug-like compounds which were approved by the United States Food and Drug Administration (FDA) for clinical trials.

#### Principal Component Analysis and Correlation Analysis

To evaluate the chemical distribution of antidepressant-related compounds in XYS, we used principal component analysis (PCA) for physicochemical-related parameters (MW, ALogP, nHDon, and nHAcc) though the prcomp function in R studio software. Therefore, known antidepressant drug/drug-like compounds from cMap, DrugBank, and active ingredients from XYS were also analyzed by PCA. Furthermore, the correlation of ADME properties of these compounds was also analyzed. Pearson r coefficients were calculated to evaluate the correlation between the three groups.

##### Target prediction

Corresponding protein targets of each active ingredient in XYS were predicted using several databases. Predicted targets from TCMSP were extracted and converted to target names using UniProtKB (http://www.uniprot.org) (Emmanuel et al., 2016). Then, PubChem, STITCH (Kuhn et al., 2012), and PharmMapper were used to obtain potential targets. The prediction results from the PharmMapper server (http://lilab.ecust.edu.cn/pharmmapper/) were based on the pharmacophore model, and this server is a large, in-house collection of pharmacophore databases extracted from all targets in TargetBank, DrugBank, BindingDB, and PDTD (Wang et al., 2017). Target information was set to Homo sapiens and aggregated for further analysis.

### Network Construction

#### Construction of an Active Compound-Targets Network for XYS

To clarify the relationship between active compounds in XYS (C) and depression-related targets, depression-related genes were retrieved from the OMIM database (Ada et al., 2005), DisGeNET database (Piñero et al., 2015), TTD database (Zhu et al., 2010), and GSE12654 gene chip. Intersections of disease (depression)-related genes (DGs) with the above predicted targets were determined to create an XYS antidepressant C-DTs network. This network was constructed and visualized using Cytoscape 3.4.0 software.

#### Comprehensive Analysis

##### Functional enrichment analysis of XYS related targets

To analyse targets groups of active compounds in XYS, we used enrichment analysis, as this method can effectively increase the reliability of the identification of biological phenomena, resulting in meaningful annotation information. (i) GO annotation: In this study, “clusterProfiler” package in R studio was used for GO (Gene Ontology) enrichment analysis. “ClusterProfiler” is based on multiple resources, and also serves as a user-friendly enrichment tool with integrated gene cluster analysis (Yu et al., 2012). This R package provides for GO gene annotation enrichment analysis, including cellular component (CC), molecular function (MF), and biological process (BP) analyses. *P* < 0.05 were considered statistically significant. (ii) KEGG pathway analysis: To explore potential biological mechanisms of predicted targets, the “clusterProfiler” R package was used to annotate Kyoto Encyclopedia of Genes and Genomes (KEGG) pathways of targets genes potentially regulated by active compounds contained in XYS. In addition, significantly enriched KEGG pathways should contain at least three genes, and have *P* < 0.05.

##### Interactions between targets in the C-DTs network

Protein–protein interaction data analysis is an important mechanism to characterize the molecular basis of disease. Interactions between each target in the C-DTs network which could be regulated by XYS were searched using the STRING website, which is a comprehensive prediction server (Szklarczyk et al., 2017). Interaction scores should be more than 0.9 to indicate appropriate confidence in identified protein interactions. Using this tool, it is feasible to discover targets related to active components of XYS based on protein network.

#### Key Active Compounds Screening and Evaluation

##### Key active compounds screening

To evaluate the antidepressant role of each active compound in XYS, hub DGs were identified using four different analysis methods in “cytoHubba” of cytoscape 3.4.0 software (Chin et al., 2014). The potential key active compounds in XYS for treatment of depression were incorporated into a Sankey diagram to reveal relationships between “hub genes - active compounds.”

##### UPLC–Q/TOF-MS analysis

The test samples of XYS according to the prescription proportion of ‘Chinese Pharmacopeia’ (AS: PN: BR: AMR: PR: ZRR: GRH: MH = 5: 5: 5: 5: 5: 5: 4: 1) were provided by Jiuzhitang Co., Ltd. (Commission, 2015). And the fine powder was carried out by crushing and mixing all herbs. Next, we accurately weighted 1 g of above powder and added 50% methanol to dissolve, then using ultrasonic treatment at room temperature for 30 min. After centrifuging at 13,000 rpm for 10 min, we injected in supernatant (2 μL) for further UPLC-Q/TOF-MS analysis.

The information of equipment used in the UPLC-Q/TOF-MS analysis was detailed described in our previous research (He et al., 2018). The universal mixed mobile phase composed of water (solvent A) and acetonitrile (solvent B), both of which contain 0.1% formic acid (v/v). Gradient elution system used a flow rate of 0.4 mL/min, and adopted as follow: 5% B, 0–0.5 min; 5–80% B, 0.5–10 min; 80–100% B, 10–12 min; 100% B, 12–13 min; 100–5% B, 13–14 min; 5% B, 14–15 min. The injection volume was set as 2 μL and under 280 nm wavelength to detect. Mass spectral data were collected in Centroided. And the purity of all reference standards was above 98% that analyzed by HPLC (High performance liquid chromatography).

#### Experiments and Molecular Evaluation

##### Cell culture

Differentiated PC12 cells were employed in the experiments. RPIM 1640 basic medium supplemented with 5% fetal bovine serum (Gibco, Rockville, MD, USA), 10% Horse serum (Gibco, Rockville, MD, USA), and 1% antibiotic mixture comprising penicillin and streptomycin (Gibco, Rockville, MD, USA). Cells were under the 5% CO_2_ in a humidified incubator (Thermo, USA) at 37°C. NGF (nerve growth factor, 50 ng/ml, 48 h) was used for PC12 differentiation.

##### Cell viability assay

The effect of paeoniflorin reverse neurotoxicity from corticosterone, cell viability was determined by the MTT assay (Fotakis and Timbrell, 2006). Briefly, PC12 cells were seeded into 96-well plate with the concentration of 5 × 10^3^/100 μl, then incubated the cells under the conventional culture condition overnight. After 24 h treatment that with the mixture of 200μM CORT plus different concentrations of paeoniflorin (10 μM, 20 μM, 40 μM), 10 μl MTT (0.5 mg/ml) was added to each well, followed by 4 h of incubation at 37°C. Then carefully remove the supernatant, and 100 μl DMSO was added to each well to dissolve the formazan crystal. The absorbance was recorded at 570 nm by microtiter plate reader. Cell viability was calculated as a percentage of the untreated or vehicle-treated control and averaged from three independent experiments.

##### Western blot analysis

Total protein was extracted from the PC-12 cells in different groups. Briefly, the collected were immediately lysed in RIPA lysis buffer containing PMSF and phosphatase protein inhibitor (Beyotime Biotechnology, Jiangsu, China). The protein lysates separated by 10% SDS-PAGE gel were electrophoretically transferred to polyvinylidene difluoride (PVDF) membranes (Millipore, Billerica, MA, USA). The following specific primary antibodies were used for incubate membranes overnight at 4°C: anti-GAPDH (Beijing BioDee Biotechnology Co., Ltd.), anti-AKT (Cell Signaling Technology), anti-Phospho-AKT (Cell Signaling Technology). After probed with a 1:5000 dilution of horseradish peroxidase (HRP) conjugated secondary antibody (Beijing BioDee Biotechnology Co., Ltd.), the labeled proteins were detected by using enhanced chemiluminescence (ECL) detection.

##### Molecular docking evaluation

Furthermore, AutoDock 4.0 was used to evaluate the hub genes with active compounds, which is widely used for the study of bound conformation and binding free energy of ligand to the protein (Forli et al., 2016).

### Statistical Analysis

Data are presented as means ± standard deviation. The standard two-tailed student's t-test was used for analysis and *P* < 0.05 were considered significant.

## Results

### Bioactive Compounds in Xiaoyao San

Bioactive ingredients in XYS and corresponding ADME information were extracted from the TCMSP data server by filtering by drug-like threshold (OB > 30% and DL > 0.18). Several of the herbal medicines in XYS contain the same active ingredients, such as quercetin, a natural flavonoid, which is present in BR and in GRH. In addition, stigmasterol is present in AS, RB and ZRR. In total, 149 active compounds were identified in XYS, including 12 in PN, 2 in AS, 18 in BR, 15 in PR, 7 in AMR, 91 in GRH, 5 in ZRR, and 10 in MH. Detailed information regarding these molecules is summarized in Table S1.

**Figure 1:**
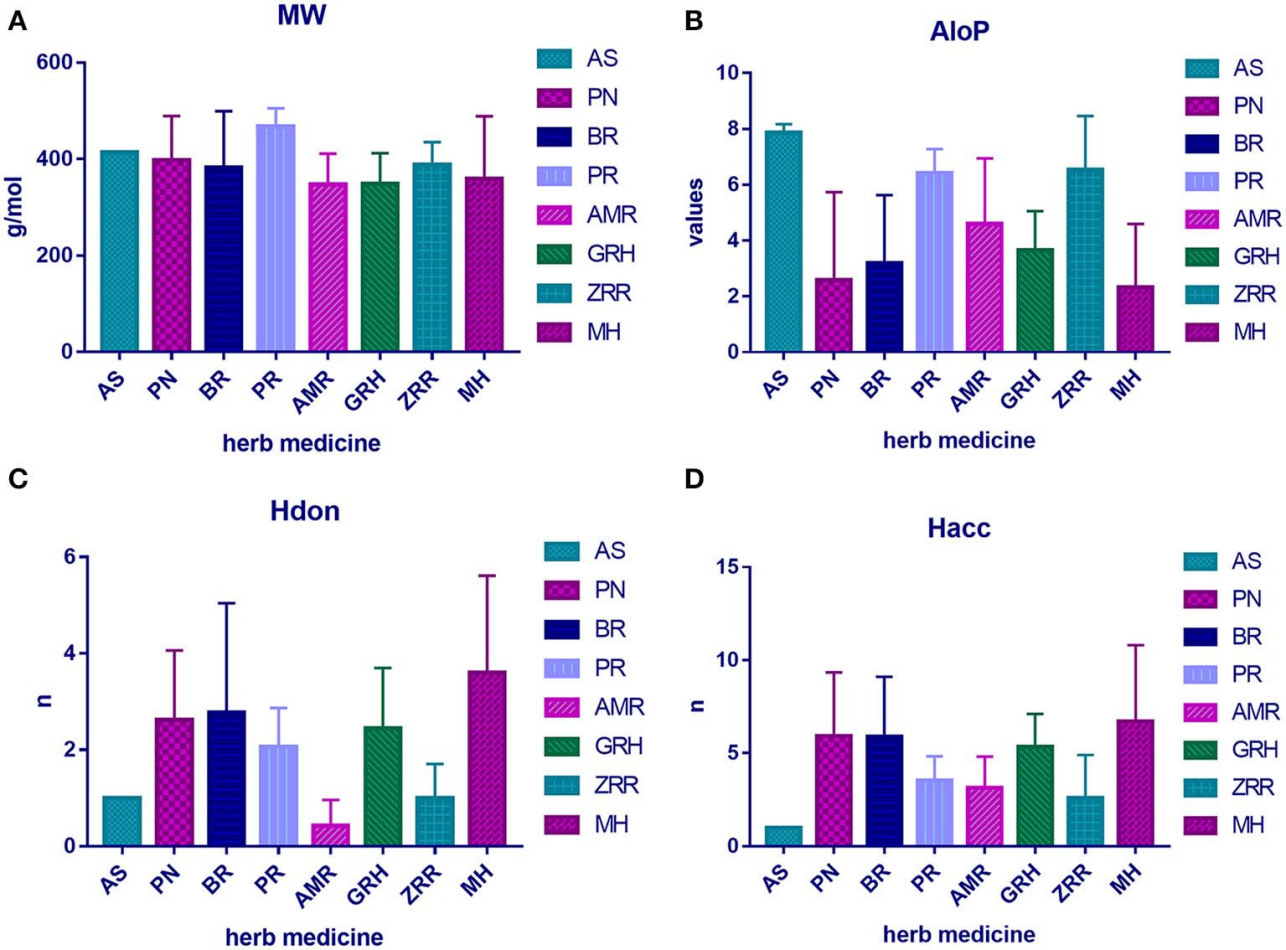
The physicochemical property of bioactive compounds from each herb in XYS. **(A)** physicochemical diversity including molecular weight (MW); **(B)** the value of partition coefficient between octanol and water (ALogP); **(C)** number of possible hydrogen-bond donors (Hdon); **(D)** number of hydrogen-bond acceptors (Hacc).

To determine the molecular diversity of the constituents of each herbal medicine, the constituents were evaluated based on four significant physicochemical properties (Figures 1A–D, Table 1): MW, ALogP, nHdon, and nHacc. (i) MW index indicated that PR had the highest average MW of bioactive components (467.78 ± 37.66), and RG (348.41 ± 63.69) had the lowest average MW of active components. (ii) Bioactive components in AS had the highest average ALogP value (7.86 ± 0.31), and MHH constituents had the lowest average ALogP value (2.32 ± 2.27). (iii) HM had the highest average number of nHdon (3.60 ± 2.01), and RAM (0.43 ± 0.53) had the lowest average number of nHdon. (iv) The herbal constituents with the highest and lowest average number of hydrogen-bond acceptors were MHH and AS, respectively.

**Table 1:** The average physicochemical value of each herb.

	**MW**	**AloP**	**Hdon**	**Hacc**
AS	413.78 ± 1.43	7.86 ± 0.31	1.00 ± 0	1.00 ± 0
PN	398.06 ± 91.19	2.58 ± 3.15	2.62 ± 1.45	5.92 ± 3.43
BR	382.10 ± 117.32	3.20 ± 2.43	2.78 ± 2.26	5.89 ± 3.33
PR	467.78 ± 37.66	6.43 ± 0.85	2.07 ± 0.79	3.53 ± 1.30
AMR	347.24 ± 64.13	4.60 ± 2.34	0.43 ± 0.53	3.14 ± 1.68
GRH	348.41 ± 63.69	3.66 ± 1.40	2.45 ± 1.25	5.33 ± 1.78
ZRR	388.88 ± 4.29	6.53 ± 1.92	1.00 ± 0.71	2.60 ± 2.30
MH	358.59 ± 130.19	2.32 ± 2.27	3.60 ± 2.01	6.70 ± 4.11

### Identified Antidepressant Drugs and PCA

We retrieved the publicly available microarray dataset GSE12654 from GEO, which included frozen brain tissues of 11 humans with depression and 15 individuals without depression (Iwamoto et al., 2004). After applying thresholds log|FC| >1.2 and *P* < 0.05 using “limma” in R studio, a total of 29 significantly differentially expressed genes between depressed and normal patients were identified. Fourteen genes were down-regulated, and 15 genes were up-regulated (Table S2).

**Figure 2:**
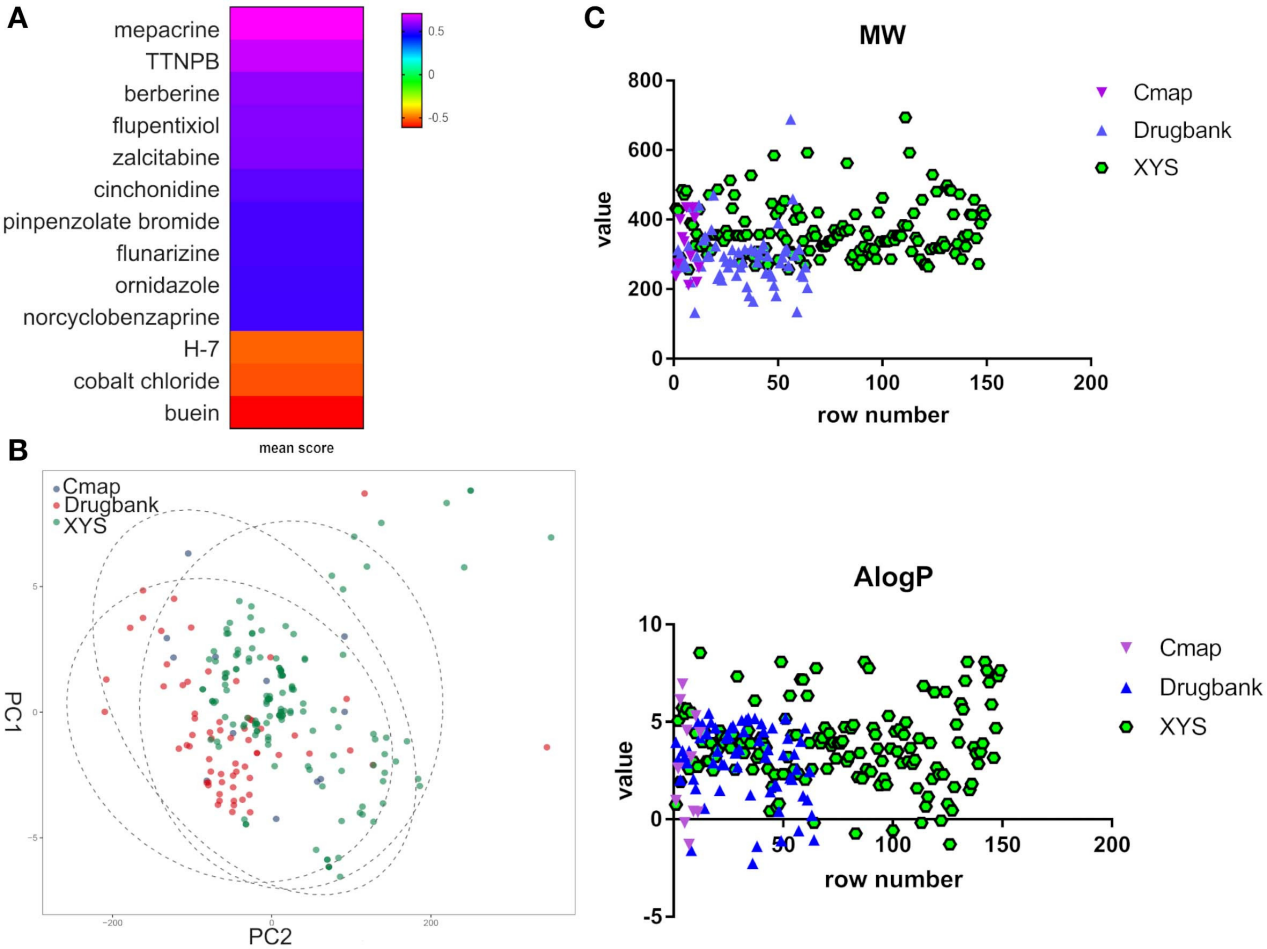
**(A)** Heatmap for mean scores of cMap antidepressant compounds. The color represents different relative connection mean score. **(B)** PCA analysis for physicochemical properties of antidepressant potential molecules. Each node indicates a compound, cMap (blue), Drugbank (red), XYS (XYS), the circle means 95%. **(C)** the correlation analysis for ADME properties of each group (the property of Hdon and Hacc have not shown).

Furthermore, to evaluate the relationship between ingredients form XYS and other known-compounds, we first identified the known-compounds which contain anti-depression function based on the DEGs results from cMap data server. Finally, 12 significant compounds with adequate relative connection mean scores (absolute value >0.5) were identified. Of the twelve compounds, 2 had high positive connection scores, and the other ten compounds possess negative connectivity scores, indicating they can act with these dysregulated genes in depression. Figure 2A and Table 2 summarize these results. In addition, a search of the DrugBank database resulted in identification of 64 FDA-approved compounds for treatment of depression (Table 2).

**Table 2:** Antidepressant compounds from cMap and Drugbank.

**Name**	**CAS**	**MW**	**AlogP**	**Hdon**	**Hacc**		
**cMap**						**Mean score**	***p*-value**
Cobalt chloride	7791-13-1	237.92	0.95	6	8	−0.825	4E-05
Butein	487-52-5	272.256	2.61	4	5	0.628	0.0002
Mepacrine	83-89-6	399.96	6.13	1	4	0.65	0.0015
TTNPB	71441-28-6	348.49	6.93	1	2	−0.63	0.0036
Berberine	2086-83-1	336.37	−0.2	0	4	0.444	0.0065
Flupentixol	2709-56-0	434.521	4.56	1	7	−0.58	0.0122
Zalcitabine	7481-89-2	211.221	−1.3	2	3	0.586	0.0137
Cinchonidine	485-71-2	294.398	3.2	1	3	−0.47	0.0189
Pipenzolate-bromide	125-51-9	434.37	0.41	4	6	−0.414	0.0192
Flunarizine	40218-96-0	404.51	5.3	0	4	−0.431	0.0204
Ornidazole	16773-42-5	219.625	0.37	1	4	−0.459	0.0208
Norcyclobenzaprine	303-50-4	261.37	4.43	1	1	0.51	0.0221
**DRUGBANK**							
Setiptiline	57262-94-9	261.368	3.96	0	1		
Reboxetine	71620-89-8	313.4	3.1	1	4		
Minaprine	25905-77-5	298.3828	2.03	1	5		
Mianserin	24219-97-4	264.3648	3.52	0	2		
Demexiptiline	24701-51-7	278.35	3.21	1	3		
Mirtazapine	85650-52-8	265.3529	2.9	0	3		
Escitalopram	128196-01-0	324.3919	3.5	0	3		
Oxitriptan	4350-9-8	220.2246	−1.6	4	4		
Tianeptine	72797-41-2	436.952	2.07	2	5		
Tranylcypromine	155-09-9	133.19	1.58	1	1		
Dimetacrine	4757-55-5	294.4338	4.96	0	2		
Vilazodone	163521-12-8	441.5249	4.21	2	4		
Desipramine	50-47-5	266.38	4.9	1	2		
Sulpiride	15676-16-1	341.43	0.57	2	5		
Adinazolam	37115-32-5	351.83	4.4	0	4		
Butriptyline	35941-65-2	293.45	5.44	0	1		
Dosulepin	113-53-1	295.44	4.98	0	1		
Trazodone	19794-93-5	371.86	2.9	0	4		
Nefazodone	83366-66-9	470	4.7	0	5		
Citalopram	59729-33-8	324.39	3.5	0	3		
Isocarboxazid	59-63-2	231.25	1.49	2	3		
Agomelatine	138112-76-2	243.3	2.83	1	2		
Pirlindole	60762-57-4	226.3	2.8	1	1		
Trimipramine	739-71-9	294.43	4.2	0	2		
Doxepin	1668-19-5	279.38	4.29	0	2		
Protriptyline	438-60-8	263.3767	4.7	1	1		
Imipramine	50-49-7	280.41	4.8	0	2		
Amoxapine	14028-44-5	313.78	3.4	1	3		
Venlafaxine	93413-69-5	277.4	2.69	1	3		
Bupropion	34911-55-2	239.74	3.6	1	2		
Sertraline	79617-96-2	306.23	5.1	1	1		
Nortriptyline	72-69-5	263.3767	4.51	1	1		
Maprotiline	10262-69-8	277.4	5.1	1	1		
Clomipramine	303-49-1	314.852	5.19	0	2		
Toloxatone	29218-27-7	207.229	1.26	1	3		
L-Tyrosine	60-18-4	181.19	−2.3	3	4		
Chlorprothixene	113-59-7	315.86	5.18	0	1		
L-Phenylalanine	63-91-2	165.19	−1.4	2	3		
Fluoxetine	54910-89-3	309.33	4.05	1	2		
Amitriptyline	50-48-6	277.4	4.92	0	1		
Vortioxetine	508233-74-7	298.45	4.51	1	2		
Fluvoxamine	54739-18-3	318.33	3.2	1	4		
Paroxetine	61869-08-7	329.37	3.6	1	4		
Levomilnacipran	96847-54-0	246.35	1.42	1	2		
Pizotifen	15574-96-6	295.44	4.7	0	1		
Milnacipran	92623-85-3	246.348	1.72	1	2		
Viloxazine	46817-91-8	237.3	1.71	1	4		
Pramipexole	104632-26-0	211.33	0.4	2	3		
Acamprosate	77337-76-9	181.21	−1.1	2	4		
Flibanserin	167933-07-5	390.4	3.32	1	3		
Cyclobenzaprine	303-53-7	275.3874	5.2	0	1		
Penbutolol	38363-40-5	291.428	4.15	2	3		
Alprazolam	28981-97-7	308.765	2.12	0	3		
Bromazepam	1812-30-2	316.15	2.05	1	3		
Pipradrol	467-60-7	267.37	3.36	2	2		
Fosnetupitant	1703748-89-3	688.61	2.57	1	6		
Levomefolic acid	31690-09-2	459.4558	−0.6	7	12		
Duloxetine	116539-59-4	297.42	4	1	2		
Phenelzine	51-71-8	135.19	1.2	2	2		
Rotigotine	99755-59-6	315.48	4.7	1	2		
Moxonidine	75438-57-2	241.677	1.01	2	6		
Carbamazepine	298-46-4	236.27	2.45	1	1		
Desvenlafaxine	93413-62-8	263.38	0.21	2	3		
L-Tryptophan	73-22-3	204.2252	−1.1	3	3		

PCA was performed to characterize the physicochemical parameters (MW, ALogP, nHDon, and nHAcc) of these antidepressant drugs/compounds. PC1 and PC2, as shown in Figure 2B and Table S3, showed a distribution of different color nodes that flocked together, indicating extensive overlap among the three groups of ingredients in XYS, and the antidepressant compounds determined by cMap and Drugbank. Furthermore, the significant positive correlation between these compounds of three groups (Figure 2C, Table S3). In ADME properties, Pearson *r* value is 0.3404 (*P* < 0.0001) for MW, AlogP (*r* = 01969, *P* = 0.003), Hdon (*r* = 0.3018, *P* < 0.0001), Hacc (*r* = 0.351, *P* < 0.0001). These results indicated that bioactive compounds from XYS are highly correlated with known antidepressant compounds in molecular properties, and could as potential drug therapies for depression treatment.

### Targets of Xiaoyao San

An *in silico* target screening approach was performed to identify the putative targets of each bioactive component of XYS. Predictive tools were used, including the TCMSP server, STITCH, and PharmMapper data servers. In total, 446 targets were identified for 149 bioactive compounds (9 compounds did not receive predictions), and many compounds were predicted to target the same proteins, such as Monoamine Oxidase B (gene symbol: MAOB). Depression-related putative targets were obtained from Therapeutic Target, DrugBank, OMIM, and DisGeNET databases, and DEGs resulting from analysis of GSE12654. Finally, 121 bioactive compounds in XYS which associated with 99 depression-related targets were identified. Multiple therapeutic targets were mediated by active ingredients of XYS, such as IL2, IL4, IL6, IL10, and STAT3, which are involved in immune and inflammatory responses closely associated with depression. For example, STAT3 (Signal transducer and activator of transcription 3), a component of the JAK/STAT signaling pathway, influences the fate and function of brain cells.

### Construction of a Compound-Depression Target Network (C-DT)

**Figure 3:**
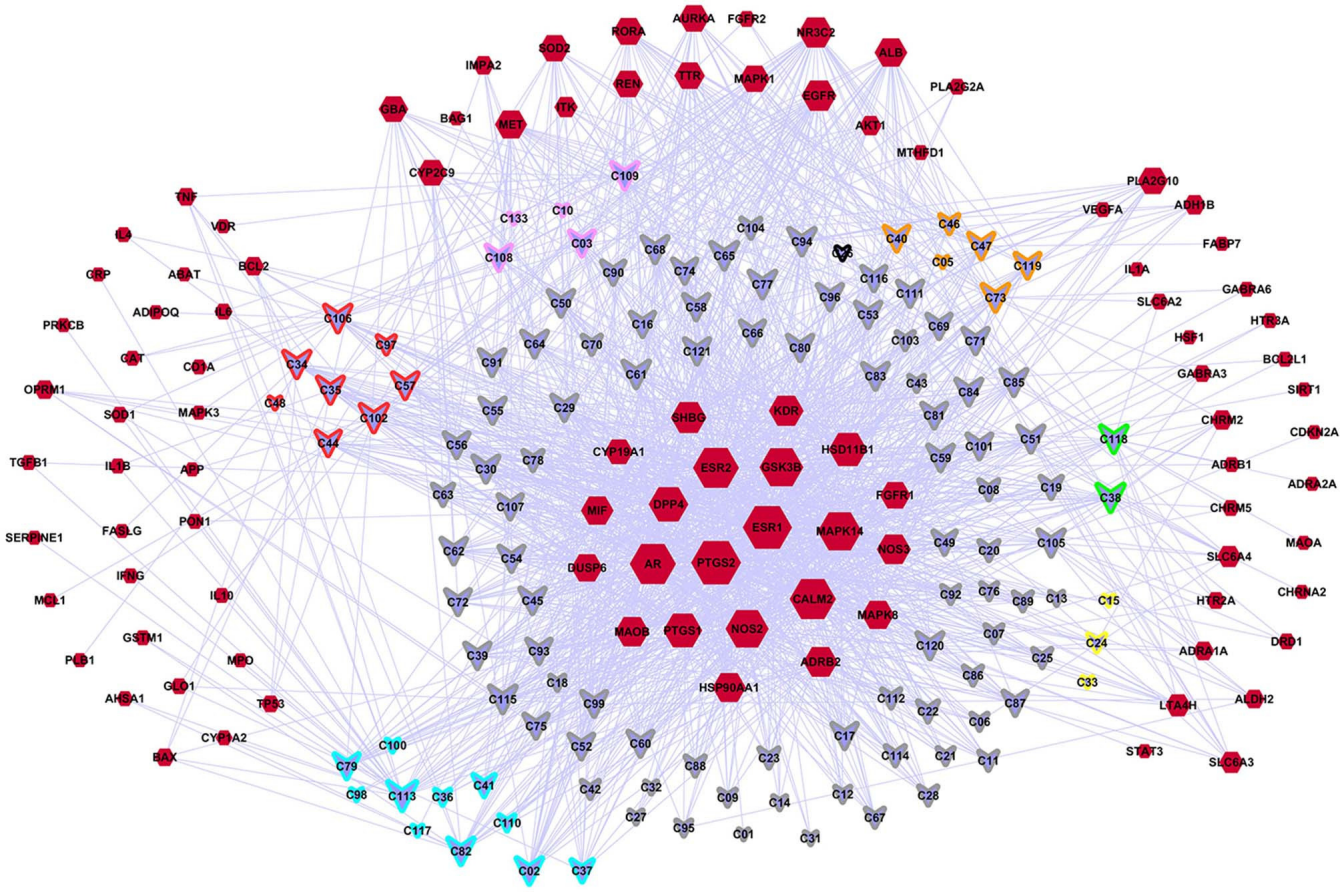
The C-DTs network. Nodes with red means depression related targets. Lavender means bioactive compounds from XYS, border color means different herbal (red: MH; orange: PR; yellow: AMR; green: AS; cyan: BR; gray: GRH; pink: PN; black: ZRR; some compounds exist in many kinds of herbal, they are randomly assigned to the corresponding colors of these herbal). And the size of each node indicate the number of connections.

A C-DT network was constructed (Figure 3) for further biological analysis. This network contained information regarding complex relationships between all bioactive compounds in XYS and their depression-related targets. There were 220 nodes in the C-DT network composed of 121 active ingredients and 99 depression targets, connected by 1583 interactions, the details were listed in Table S4. As shown in Figure 3, node size indicated the number of connections calculated using “NetworkAnalyzer,” an analytical tool in cytoscape 3.7.0.

### Enrichment Analysis of the C-DT Network

#### GO and KEGG Pathway Analysis

**Figure 4:**
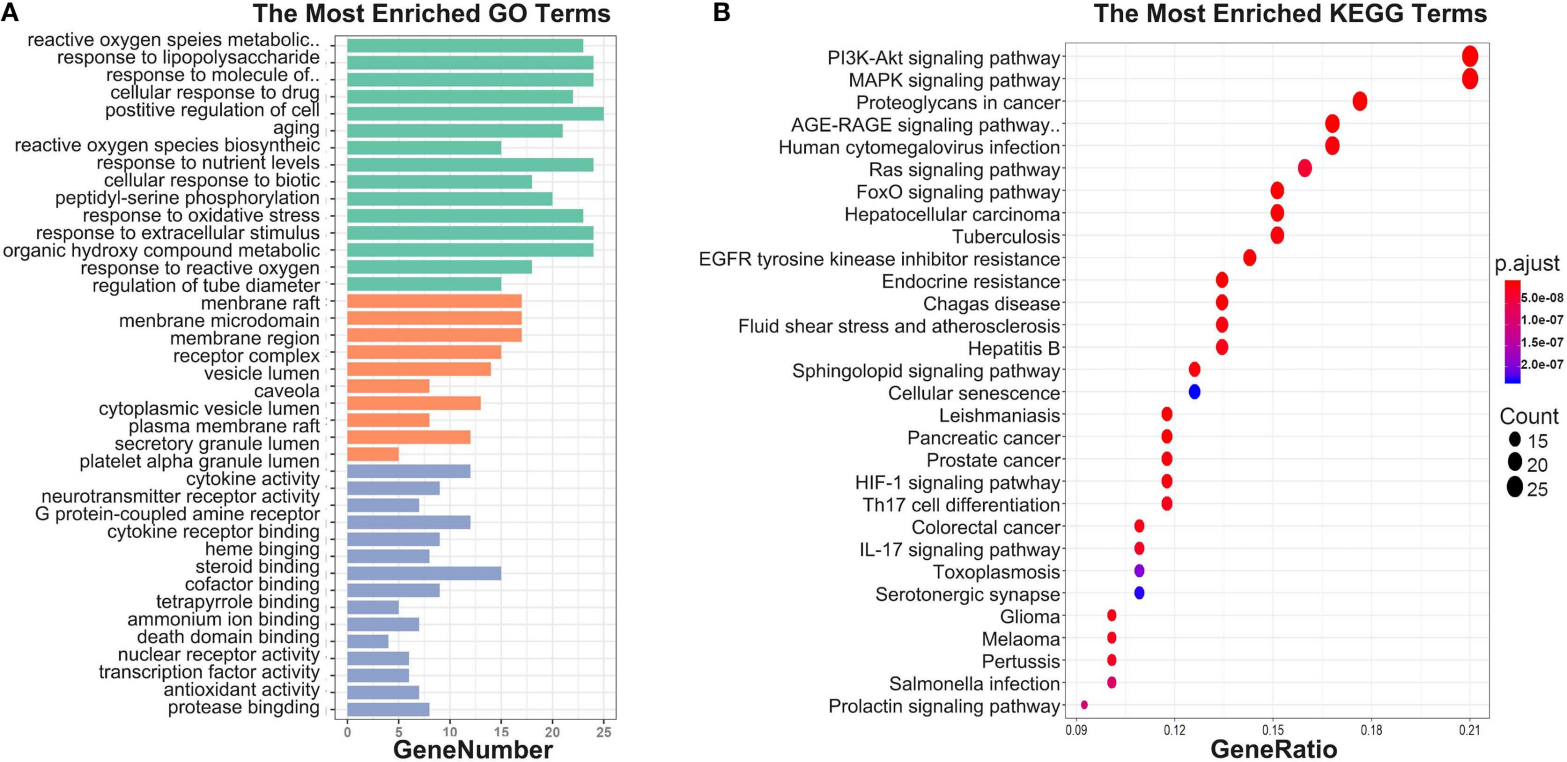
**(A)** GO enrichment. Each bar indicates a CC/MF/BP term, color means enriched type (green color present CC, orange present MF, light blue present BP), and abscissa represents the number of enriched genes. **(B)** KEGG enrichment. Dotplot for KEGG pathway, the size of each node indicates enriched counts. abscissa represents the enriched gene ratio. Color means enriched adjust *P*-value.

To investigate biological functions associated with this interaction network relative to treatment of depression, we performed GO and KEGG pathway enrichment analyses using the “clusterprofile” R package. Three GO categories were analyzed, which included CC, MF, and BP. The top 15 GO enrichment results for each category are listed in Table 3. Direct outcomes are shown in Figure 4A. These targets were significantly involved in formation of neuronal receptor and cell, which are directly related to generation of brain cells and tissue. Molecular functions involved activity and binding of various molecules, neurotransmitter receptors, cytokines, transmembrane receptor protein kinases, and nuclear receptor, as well as cytokine receptor binding, phoshphatase, and steroid. Moreover, combined BP results indicated that XYS may regulate depression-related biological processes, such as “regulation of cell migration,” “reactive oxygen species metabolic process,” and “aging” to exert antidepressant effects.

**Table 3:** GO enrichment analysis.

**ID**	**Description**	**Count**	**q value**
**BP**			
GO:0030335	positive regulation of cell migration	24	3.57E-14
GO:1901615	organic hydroxy compound metabolic process	24	3.70E-14
GO:0032496	response to lipopolysaccharide	23	1.75E-16
GO:0002237	response to molecule of bacterial origin	23	3.87E-16
GO:0031667	response to nutrient levels	23	6.90E-14
GO:0009991	response to extracellular stimulus	23	1.68E-13
GO:0072593	reactive oxygen species metabolic process	22	3.04E-17
GO:0006979	response to oxidative stress	22	1.45E-13
GO:0035690	cellular response to drug	21	3.64E-14
GO:0018108	peptidyl-tyrosine phosphorylation	21	1.39E-13
**MF**			
GO:0019902	phosphatase binding	11	2.94E-07
GO:0005057	NA	11	2.94E-07
GO:0005125	cytokine activity	12	2.94E-07
GO:0030594	neurotransmitter receptor activity	9	9.10E-07
GO:0019903	protein phosphatase binding	9	1.03E-06
GO:0004702	NA	9	1.03E-06
GO:0008227	G protein-coupled amine receptor activity	7	1.03E-06
GO:0005496	steroid binding	8	1.33E-06
GO:0005126	cytokine receptor binding	12	1.33E-06
GO:1901338	catecholamine binding	5	2.33E-06
**CC**			
GO:0045121	membrane raft	16	4.00E-10
GO:0098857	membrane microdomain	16	4.00E-10
GO:0098589	membrane region	16	4.57E-10
GO:0043235	receptor complex	15	4.60E-08
GO:0031983	vesicle lumen	14	9.61E-08
GO:0060205	cytoplasmic vesicle lumen	13	7.21E-07
GO:0034774	secretory granule lumen	12	3.03E-06
GO:0005901	caveola	7	3.90E-06
GO:0044853	plasma membrane raft	7	1.67E-05
GO:0031093	platelet alpha granule lumen	5	0.0004881

KEGG signaling pathway results are shown in Figure 4 and Table 4. The top enriched terms were related to several critical pathways associated with disease, such as “PI3K-Akt signaling pathway,” “MAPK signaling pathway,” and “neuroactive ligand-receptor interaction” (Figure 4B and Figure S1). These findings demonstrated complex pathological features of depression and the potential of XYS as an antidepressant.

**Table 4:** KEGG pathway.

**ID**	**Description**	**p.adjust**	**Count**
hsa04151	PI3K-Akt signaling pathway	6.80E-08	20
hsa04010	MAPK signaling pathway	2.59E-08	19
hsa05163	Human cytomegalovirus infection	4.02E-09	18
hsa04933	AGE-RAGE signaling pathway in diabetic complications	4.07E-13	17
hsa05152	Tuberculosis	1.47E-09	17
hsa05205	Proteoglycans in cancer	2.59E-08	16
hsa04014	Ras signaling pathway	1.44E-07	16
hsa01521	EGFR tyrosine kinase inhibitor resistance	1.90E-12	15
hsa05418	Fluid shear stress and atherosclerosis	2.74E-09	15
hsa05142	Chagas disease (American trypanosomiasis)	9.13E-10	14

#### PPI Network Analysis and Screening of Key Active Ingredients

**Figure 5:**
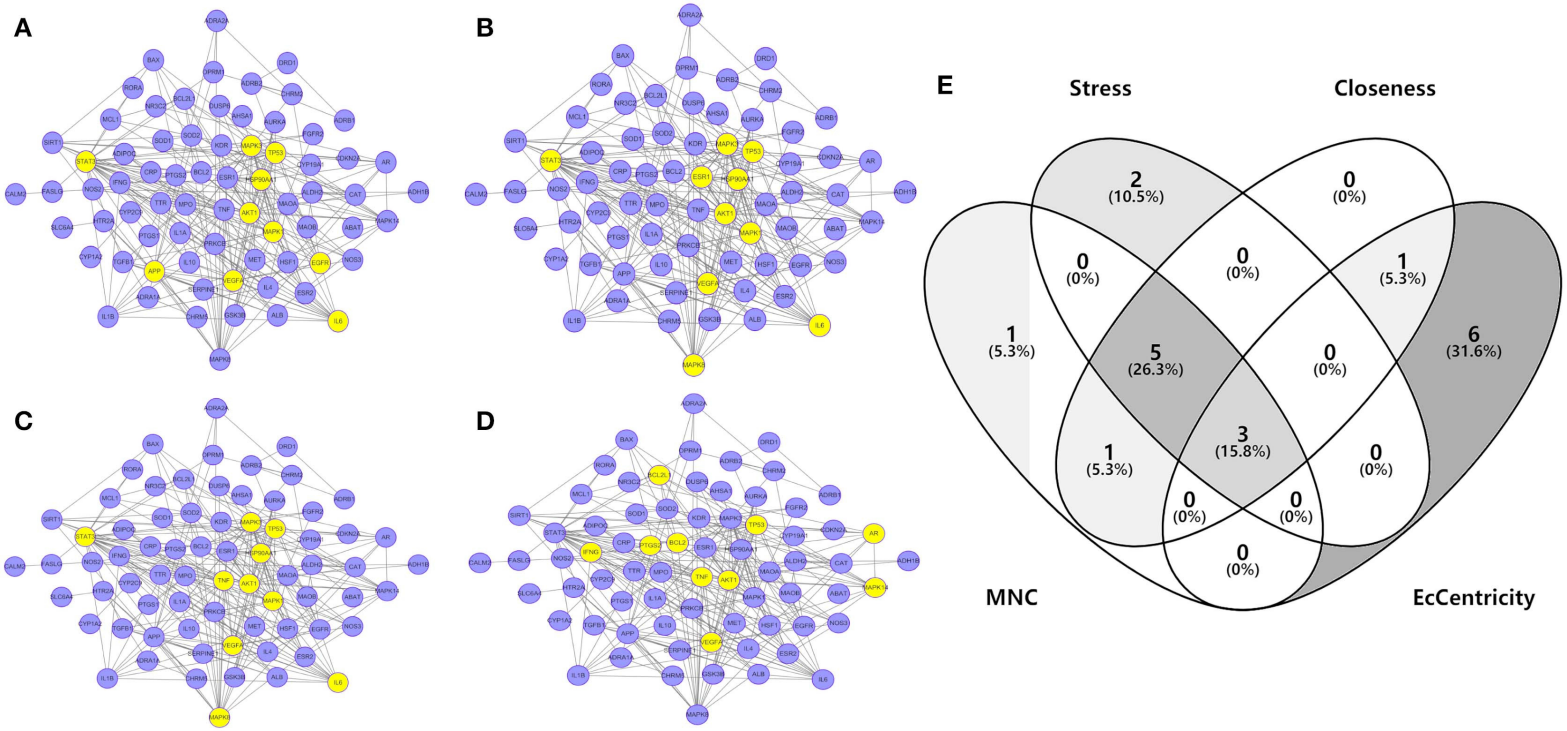
**(A–D)** PPI network analysis. Networks represent the analysis results of the based different aspect (Stress, Closeness, MNC and EcCentricity), respectively. The highlight yellow nodes are hub targets (top 10). And the light purple nodes are also highest confidence protein targets from C-DTs network. **(E)** Venn diagram. The overlapping genes were selected which as core hub targets for further analysis.

**Figure 6:**
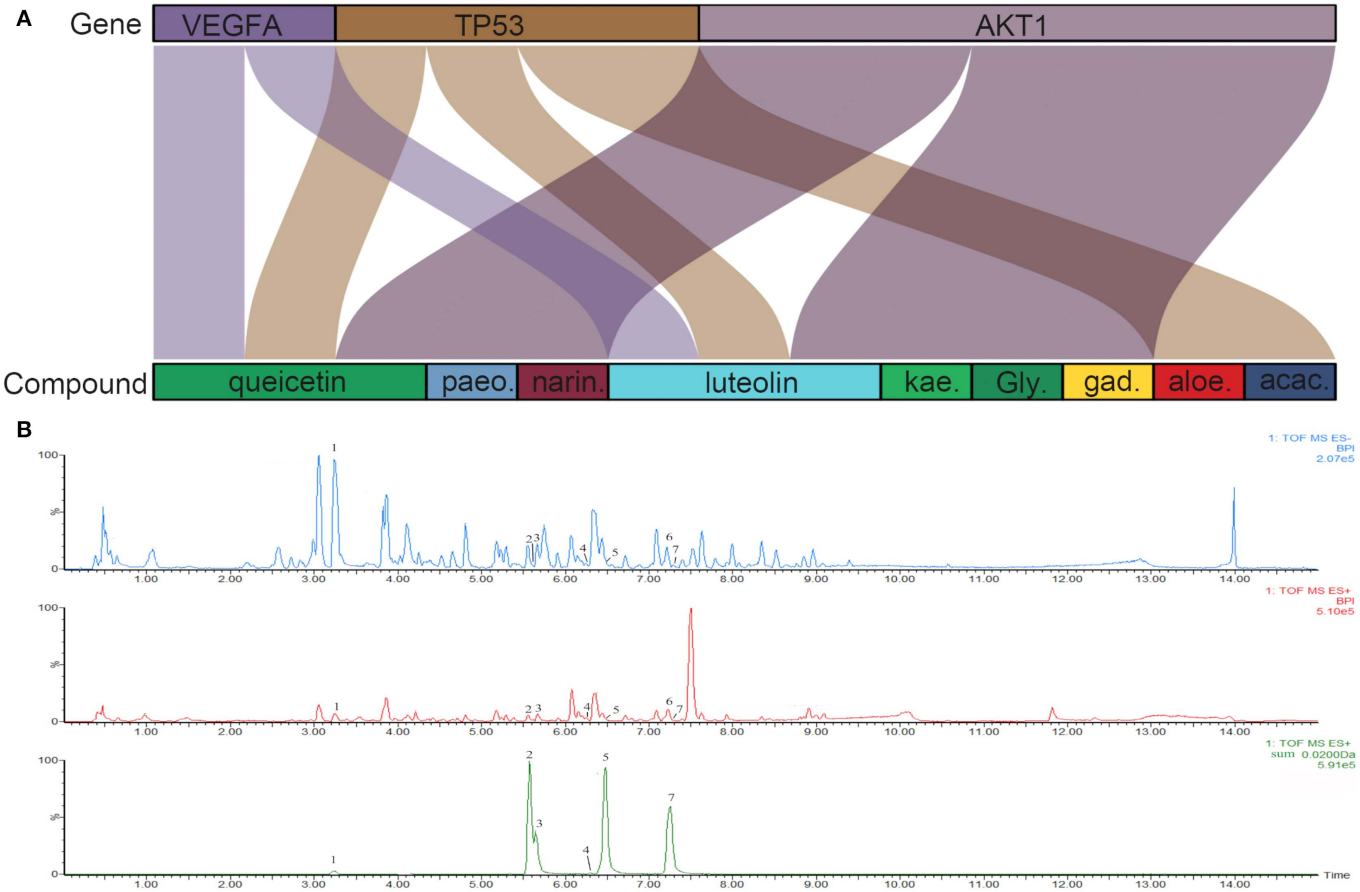
**(A)** Sankey diagram. Sankey diagram direct shown the relationship between compounds and targets. The blocks on the up represent the hub targets. And the underneath blocks indicate compounds, as follow: quercetin, luteolin, acacetin, aloe-emodin, gadelaidic acid, Glyasperin C, kaempferol, naringenin, paeoniflorin. **(B)** The chemical base peak intensity (BPI) chromatogram of key compounds characterization of XYS in positive ion mode and negative ion mode determined by UPLC-Q-TOF/MS. Identification No.:1. Paeoniflorin; 2. Kaempferol; 3. Quercetin; 4. Aloe emodin; 5. Luteolin; 6. Glyasperin C; 7. Acacetin.

To investigate the characteristics of protein targets in the C-DT network, protein–protein interactions were analyzed using the STRING online database, and the related PPI network was constructed and visualized using cytoscape software. Using confidence of 0.9 as the threshold, we found 90 nodes and 288 interactions in the PPI network. In addition, we used four different analysis methods (Stress, Closeness, MNC and EcCentricity) using the “cytoHubba” plugin to identify the top 10 hub nodes in the PPI network. Detailed results are shown in Figures 5A–D, and the top hub genes based on these four indices were summarized in a Venn diagram (Figure 5E, Table S5). Thus, 3 core genes were identified as hubs: AKT1, TP53, and VEGFA. Furthermore, based on GO and KEGG analyses, these three core genes were enriched in multiple biological processes and signaling pathways. For instance, AKT1 is involved in myelination of the peripheral nervous system, peripheral nervous system axon ensheathment, and the neurotrophin signaling pathway, all of which are closely related to depression (Barros et al., 2009; Musashe et al., 2016; Liu et al., 2018). Using these core genes, a Sankey diagram (Figure 6A) was used to facilitate shown of bioactive compounds that can act on multiple targets. Nine ingredients of XYS and their hub targets were identified, including quercetin, luteolin, acacetin, aloe-emodin, gadelaidic acid, Glyasperin C, kaempferol, naringenin, paeoniflorin.

### Identification of the Chemical Characterization of XYS

**Figure 7:**
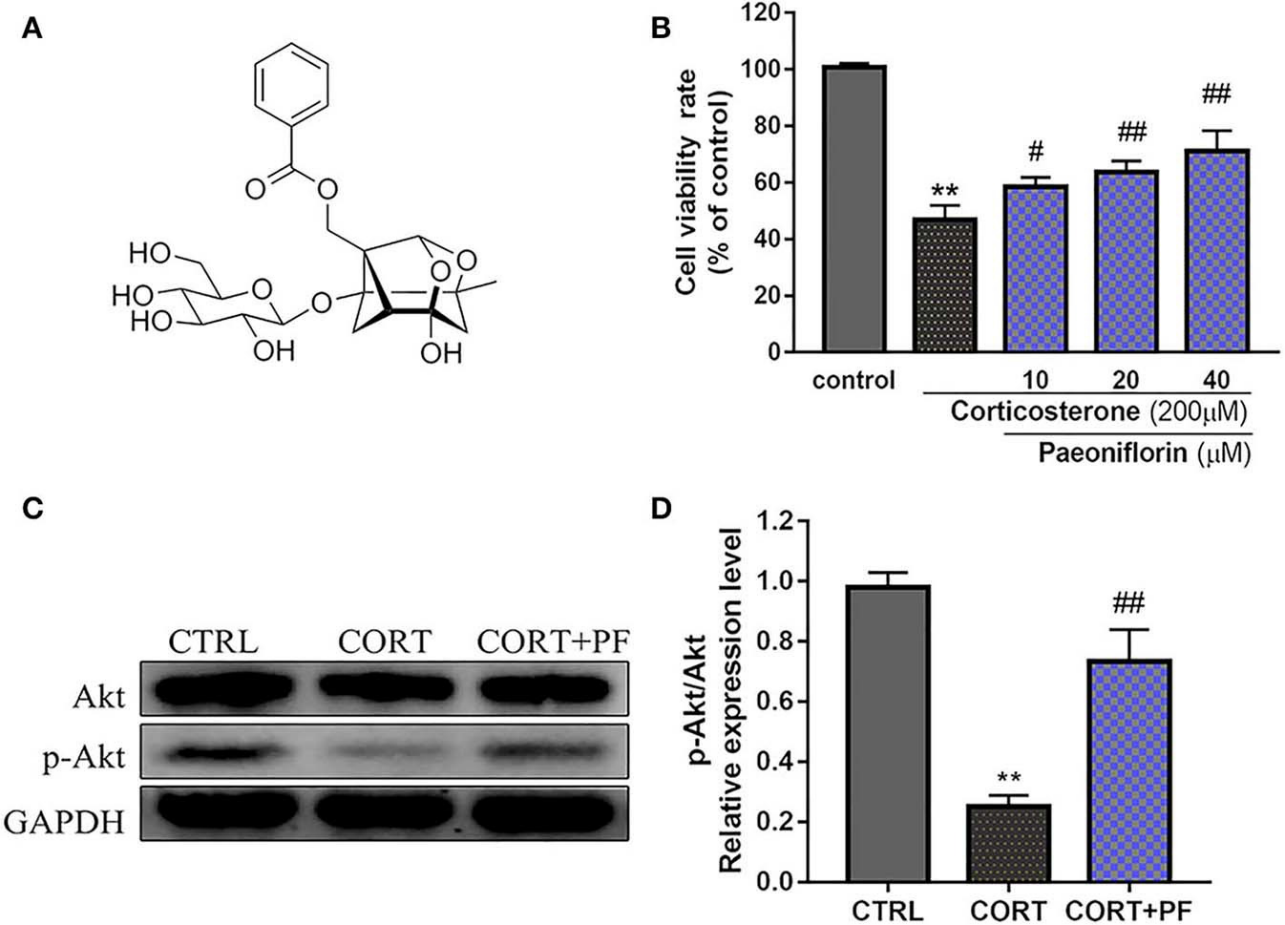
**(A)** Chemical structure of Paeoniflorin; **(B)** Viability of PC12 cells assessed using MTT assay following culture of 200μM CORT with different dosages of paeoniflorin (10, 20, 40μl/ml) for 24 h. **(C)** Western blots of p-Akt expression in different treated condition PC-12 cells. GAPDH as the loading control. **(D)** Densitometry data analysis were quantified using Image J software. Compare with control group, **P < 0.01; compare with CORT group, ^#^*P* < 0.05, ^*##*^*P* < 0.01; *n* = 3. PF, paeoniflorin; CORT, corticosterone.

As shown in Figure 6B, the chemical base peak ion (BPI) chromatogram of XYS based on the positive and negative ion modes of UPLC-Q-TOF/MS. According to the above analysis, nine key compounds of XYS were further examined. All of the key compounds from XYS, seven ingredients (paeoniflorin, quercetin, luteolin, acacetin, aloe-emodin, Glyasperin C, kaempferol) were successful identified by UPLC-Q-TOF/MS analysis, the details were listed in Table 5. Among them, we found major compounds are flavonoids, only paeoniflorin as monoterpene glycoside, and most importantly it is a principal ingredient of *Paeonia lactiflora* Pallas (Figure 7A). Thus, we next performed cellular model and investigated the neuron protected effect of paeoniflorin *in vitro*.

**Table 5:** Chemical composition of key ingredients from XYS identified by UPLC-Q/TOF-MS.

**No**.	**Time**	**Selected ion**	**Elemental composition**	**Calculated mass**	**Mass error**	**(ESI+) MS/MS fragmentation**	**(ESI-) MS/MS fragmentation**	**Identification**
1^*^	3.24	[M-H+HCOOH]^−^	C24H29O13	525.1608	1.7	319.1174, 301.1065, 197.0806, 179.0711, 151.0758, 105.0337	479.1556, 449.1453, 431.1345, 327.1080, 165.0547, 121.0291	Paeoniflorin
2^*^	5.57	[2M-H]^−^	C30H19O12	571.0877	3.2	161.0232, 153.0192	285.0414, 151.0039	Kaempferol
3^*^	5.65	[M+H]^+^	C15H11O7	303.0505	1.6	229.0497, 179.0344, 153.0188, 109.0290	603.0786, 301.0354, 151.0037, 121.0288	Quercetin
4^*^	6.29	[M+H]^+^	C15H11O5	271.0606	−0.7		269.0459	Aloeemodin
5^*^	6.48	[2M-H]^−^	C30H19O12	571.0877	−2.6	153.0192, 109.0289	285.0407	Luteolin
6	7.20	[M+H]^+^	C21H25O5	357.1702	0.6	301.1076, 221.1179, 165.0555	355.1546	Glyasperin C
7^*^	7.26	[M+H]^+^	C16H13O5	285.0763	−2.5	270.0524, 242.0575, 153.0183	283.0612, 211.0396	Acacetin

* *Accurately identified with reference standards*.

### Evaluations

To determine the protected effect of Paeoniflorin from CORT neurotoxicity, the cell viability assay was examined. The detailed MTT assay results as shown in Figure 7B, that 200 μM CORT reduced the cell viability of PC12 cells, whereas treatment 20 and 40 μM paeoniflorin were very significant able to moderate the cell viability (*P* < 0.01). Western blot analysis results showed that compared with the control cells, the expression of p-Akt in CORT stimulated cells was significantly decreased (*P* < 0.01). However, the down-regulated expression of p-Akt was significantly reversed by paeoniflorin treatment (Figures 7C,D).

**Figure 8:**
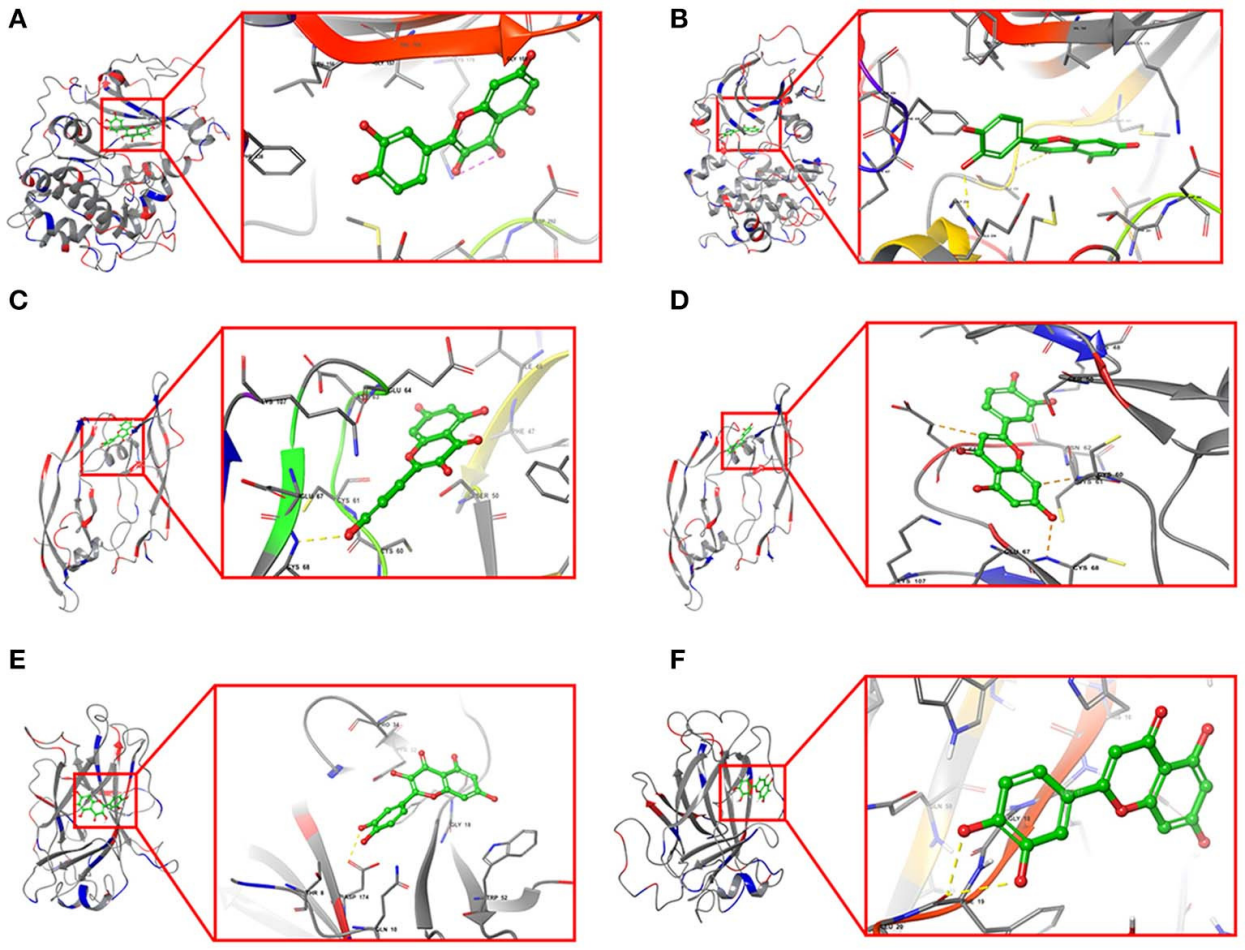
Structural model and binding site of active ingredients with hub targets. **(A)** Docking site between AKT1 with quercetin. **(B)** Docking site between AKT1 with luteolin. **(C)** Docking site between VEGFA with quercetin. **(D)** Docking site between VEGFA with luteolin. **(E)** Docking site between TP53 with quercetin. **(F)** Docking site between TP53 with luteolin.

Furthermore, quercetin and luteolin could act on all hub genes, so we further validated their molecular docking to detect and calculate the binding energy between small molecules and proteins. The best bound conformation site of quercetin and luteolin binding to proteins were shown in Figure 8, respectively. The binding energy between quercetin with AKT1 is −17.6341 kcal/mol, and luteolin with AKT1 is −12.7977 kcal/mol, that initiated both two compounds had strong binding activity with AKT1. Similarly, these two compounds contain strong binding activity with other hub targets, the binding energy between quercetin with VEGFA and TP53 (−67.4121 kcal/mol), luteolin with VEGFA and TP53 (−53.3754 kcal/mol).

## Discussion

Traditional Chinese medicine (TCM) is being more frequently chosen by patients to treat depressive and anxiety disorders. A meta-analysis reported that use of XYS as an adjuvant therapy with antidepressant drugs in 1,837 patients was more effective in improving symptoms than antidepressants alone (Zhang et al., 2011). Furthermore, previous studies indicated that XYS treatment of depression and anxiety involves several biological aspects. For instance, some psychological disorders have been associated with increased cytokine levels resulting from immune activation, known as the “cytokine hypothesis” (Sharma, 2016). Inflammatory cytokines may act as modulators of 5-HT (serotonin) receptors, and upregulate indoleamine 2,3-dioxygenase (IDO), resulting in anxiety-like disorders (Myint and Kim, 2003). The antidepressant-like effects of XYS were associated with downregulation of peripheral IL-1β, IFN-γ, TNF-α, and IL-6, and increased synthesis of tryptophan hydroxylase (TPH) and IDO, which play a role in promoting 5-HT synthesis (Jiao et al., 2019). XYS significantly inhibited TNF-α release in serum and the hippocampus through activation of the TNF-α/JAK2-STAT3 pathway, as shown in our preliminary study (Li et al., 2017). Moreover, the HPA axis and brain-derived neurotrophic factor (BDNF) have also been widely studied in the context of pathogenesis and treatment of depressive disorders (Jiang et al., 2016). Previous studies have demonstrated that XYS could reverse chronic stress-induced anxiety-like symptoms by regulating the apelin-APJ system in the hypothalamus and activity of the HPA axis (Yan et al., 2018).

Many studies have demonstrated the antidepressant effects of XYS, which involves multiple pathways associated with depression. Therefore, the complex bioactive ingredients and targets with potential antidepressant activities require clarification. Previous studies using High Performance Liquid Chromatography coupled with LTQ Orbitrap Mass Spectrometry (HPLC-LTQ-Orbitrap-MS) identified paeoniflorin, liquiritin, glycyrrhizic acid, ferulic acid, saikosaponins A and C, curcumin, and poria cocos alcohol were eight active compounds identified in XYS (Li et al., 2015). Yuzhi Zhou and his colleagues identified 4 components: Z-ligustilide, palmitic acid, atractylenolide I, and atractylenolide II in XYS using gas chromatography–mass spectrometry (GC-MS) methods (Zhou et al., 2012). In addition, network pharmacology methods have been used to predict and analyse 13 components of XYS (Gao et al., 2015). However, no comprehensive analysis of all bioactive compounds in XYS had been previously reported. Therefore, the present study adopted a comprehensive systematic network pharmacology approach for evaluation of XYS.

In this study, we identified 149 compounds in active fractions of XYS. PCA analysis and correlation analysis was used to compare these compounds with antidepressants approved by the FDA and small molecule drugs predicted by the cMap database. This was the first comparative study of drug properties of TCM formula compounds using Drugbank and a gene chip combined with cMap. Our results indicated that these 149 bioactive compounds in XYS possessed potential antidepressant properties. Therefore, we constructed a compound-depression targets (C-DTs) network, and further enriched this network using GO KEGG pathways analyses of 99 depression-related targets. CC enrichment results indicated that a considerable number of genes were involved in formation of nerves and synapses. Synapses are the fundamental structures for information transmission between neurons, and they adapt to stimuli by continuously modifying neural connections and neurological circuits. Therefore, synaptic plasticity is the main manifestation of neuroplasticity, and depression and other psychological disorders are typically associated with decreased synaptic plasticity in the hippocampus (Wainwright and Galea, 2013). Molecular functions associated with components of XYS involved activity and binding of various molecules, such as neurotransmitter receptors, cytokines, transmembrane receptor protein kinases, nuclear receptors, cytokine receptor binding, steroids, and catecholamines. Moreover, a large number of genes were significantly enriched as BP terms in regulation of aging, reactive oxygen species metabolic processes, and inflammatory response, which are associated with pathogenesis of depression. Combined BP results indicated that XYS may act via generation of nerves and synapses, alteration of neurotransmitter receptor function, and regulation of cytokines, resulting in antidepressant effects. The PI3K/Akt signaling pathway was enriched to the greatest degree, resulting in association of 20 genes. It has been wildly regarded to modulate antidepressant-like functions and contributes to synaptic plasticity and neurotransmission formation (Ludka et al., 2016). Furthermore, several metabolic pathways directly related to the nervous system such as “neuroactive ligand-receptor interaction,” “GABAergic synapse,” and “neurotrophin signaling pathway” were also enriched. Analysis of the PPI network identified hub nodes which were most closely related to other proteins: AKT1, TP53, VEGFA. These proteins were consistent with our enrichment analysis, and have been previously reported to be related to neurotrophic factors and depression.

Bioactive ingredients in XYS were classified in the following categories: volatile oils, alkaloids, flavonoids, saponins, and polysaccharides. Among these, polysaccharides can regulate intestinal flora to moderate depression though the brain-gut-microbiota axis (Cryan and Dinan, 2012; Schwalm et al., 2016), and flavonoids can cross the blood-brain barrier, directly acting on brain targets (Youdim et al., 2003). We concluded that XYS exerts its effects in many ways through the actions of many compounds and may confer advantages over the compounds by reducing drug resistance.

After identified hub targets, 9 ingredients of XYS could interact with them, and 7 of them were identified by UPLC-Q/TOF-MS analysis. As a result, we found that 3 hub genes predicted by the PPI network could be regulated by quercetin and luteolin, which are both flavonoids. Nevertheless, most of the reports of flavonoids are limited to *in vitro* experiments, and these flavonoids are common constituents in a variety of herbs. It is considered important to clarify their specific role in the treatment (Heinricha et al., 2020). Actually, many studies have verified their neuroprotective effects *in vitro* and *in vivo*. For instance, the genes regulated by luteolin could act as receptors in the central nervous system, resulting in antidepressant effects (Sasaki et al., 2013; Akinrinde and Adebiyi, 2019). Quercetin, a flavonoid, is a component of *Bupleurum Chinense* Dc. This flavonoid confers antioxidant activity and anti-inflammatory effects in the treatment of neurological diseases. A previous study reported that quercetin relieved anxiety and depression of chronic stress rats by protecting neurons from oxidation and inflammation (Mehta et al., 2017). In LPS-induced neuroinflammation of mice, quercetin still exhibited a function of eliminating inflammation through significantly reduced LPS-induced proliferation of astrocytes in the brain and decreased the expression of inflammatory factors (Khan et al., 2018). Additionally, researchers found that quercetin could inhibit neuronal apoptosis to preventing anxiety-like behaviors *in vivo* via regulating the Akt1 and ASK1/JNK3/caspase-3 expression (Pei et al., 2016). Besides, in a previous network pharmacology study, quercetin was found also as a high contribution compound of Huangqi and Huanglian for treating diabetes, participated in polypharmacological and synergistic mechanisms (Yue et al., 2017).

However, among these 7 ingredients, only paeoniflorin, a monoterpene glycoside compound, as main component of herb (*Paeonia lactiflora* Pallas) from XYS. And it is one of the quality identification standards for XYS in the Chinese Pharmacopeia. Thus, it should be a representative compound from this formula, so that the current research further analyzed its neuroprotective effect and validated its potential roles with predicted target Akt. Previous reports by several researchers had provided clues supporting the role for paeoniflorin in the protection of neuron cells and the therapeutic effects in neurological diseases (Mao et al., 2012; Cong et al., 2019). Gu et al. had demonstrated that paeoniflorin via upregulating the p-Akt expression and Bcl-2/Bax ratio to reduce neuron death in Alzheimer's disease mice (Gu et al., 2016). Recent evidence suggests that CUMS rats had improved depressive-like behavior after paeoniflorin treatment, it could be regulated by acting on the ERK-CREB signaling pathway (Zhong et al., 2018). Consist with previous findings, we found paeoniflorin can reverse the neurotoxicity produced through CORT and promote the phosphorylation of Akt. On the one hand, from the perspective of network pharmacology, the representative importance of paeoniflorin in XYS has been clarified for the first time. On the other hand, our results indicated that paeoniflorin as the XYS's representative compound which could modulate the several signaling upstream target AKT's expression, that validate the current network pharmacology prediction.

## Limitations and Conclusions

Current research based on network pharmacology which could provide a novel and systematic analysis way for the research of Chinese herbal formula. However, there are still some limitations in our article. Firstly, as we all know, components of Chinese herbal are complicated, it's doubtful whether there are synergistic or side effects caused by the interaction between compounds that is hard to clarified in the prediction and composition identification of this study. Secondly, although CORT is one of the most common methods to simulate depression *in vitro*, the pathogenesis of depression is complicated, which means that it is difficult to simulate whole pathogenic factors *in vitro*. Moreover, western blots suggested that paeoniflorin could regulate the phosphorylation of Akt, but it is only in protein expression level. The direct or indirect regulation between them would need to be further explored. Last but not least, hub genes were selected by PPI network in our study that they are usually as upstream molecules or central molecules in those important signaling pathways. However, there still exist multiple downstream proteins in pathogenesis of depression. And thus, it is important to discover and identify more compounds that interact with these downstream proteins through future works.

Overall, this study systematically summarized key active compounds in XYS and comprehensively analyzed potential mechanisms of its action and pathways. We also evaluated for representative compound paeoniflorin *in vitro*. The current findings provide a basis and new insights for XYS in the treatment of depression. Certainly, we will explore widely experiments of pharmaceutical ingredients to better understand the effects of XYS, such as component analyses, identification of drugs, and metabolic studies in animal models.

## Data Availability Statement

Publicly available datasets were analyzed in this study. This data can be found here: https://www.ncbi.nlm.nih.gov/geo/query/acc.cgi?acc=GSE12654.

## Author Contributions

NY and JC main contributed research conception and design. NY, KT, LH, WH, XL, and QM collected and processed data. NY, LG, and KT wrote sections of the manuscript. All authors contributed to manuscript revision, read, and approved the final submitted version. JC takes primary responsibility for communication with the journal and editorial office during the submission process, throughout peer review, and during publication.

### Conflict of Interest

The authors declare that the research was conducted in the absence of any commercial or financial relationships that could be construed as a potential conflict of interest.

## Supplementary Material

Figure S1 Distribution of partial targets of XYS on the Neuroactive ligand-receptor interaction signaling pathway. The red nodes are potential targets. The green nodes are relevant targets in the pathway.

Table S1 Bioactive ingredients of XYS.

Table S2 The DEmRNAs of GSE12654.

Table S3 **(A)** PCA analysis for physicochemical properties of potential antidepressant molecules; **(B)** Correlation analysis for properties between each molecular group.

Table S4 C-DTs network.

Table S5 Compounds acting on hub targets.

## Abbreviations

CAM: Complementary and alternative medicine
TCM: Traditional Chinese Medicine
XYS: Xiao-Yao-San
LSSDS: Liver stagnation and spleen deficiency syndrome
AS: *Angelica sinensis* (Oliver) Diels
PN: *Paeonia lactiflora* Pallas
BR: *Bupleurum Chinense* De Candolle
AMR: *Atractylodes macrocephala* Koidzumi
PR: *Poria cocos* (Schweinitz) Wolf
MH: *Mentha canadensis* Linnaeus
GRH: *Glycyrrhiza uralensis* Fischer
ZRR: *Zingiber officinale* Roscoe
OB: oral bioavailability
DL: drug-likeness
DEGs: differentially expressed genes
PCA: principal component analysis
GO: Gene Ontology
CC: cellular component
MF: molecular function
KEGG: Kyoto Encyclopedia of Genes and Genomes
MW: molecular weight
ALogP: the value of partition coefficient between octanol and water
nHdon: the number of possible hydrogen-bond donors
nHacc: and the number of hydrogen-bond acceptors
BDNF: brain-derived neurotrophic factor
UPLC–Q/TOF-MS: ultra-high performance liquid chromatography-quadrupole time-of-flight mass spectrometry
CORT: corticosterone
PF: paeoniflorin.
