# Supplementary figures and images for: An Integrated Pharmacology-Based Analysis for Antidepressant Mechanism of Chinese Herbal Formula Xiao-Yao-San
Source: Front Pharmacol. 2020 Mar 18;11:284. doi: 10.3389/fphar.2020.00284 (PMC7094752; doi:10.3389/fphar.2020.00284)

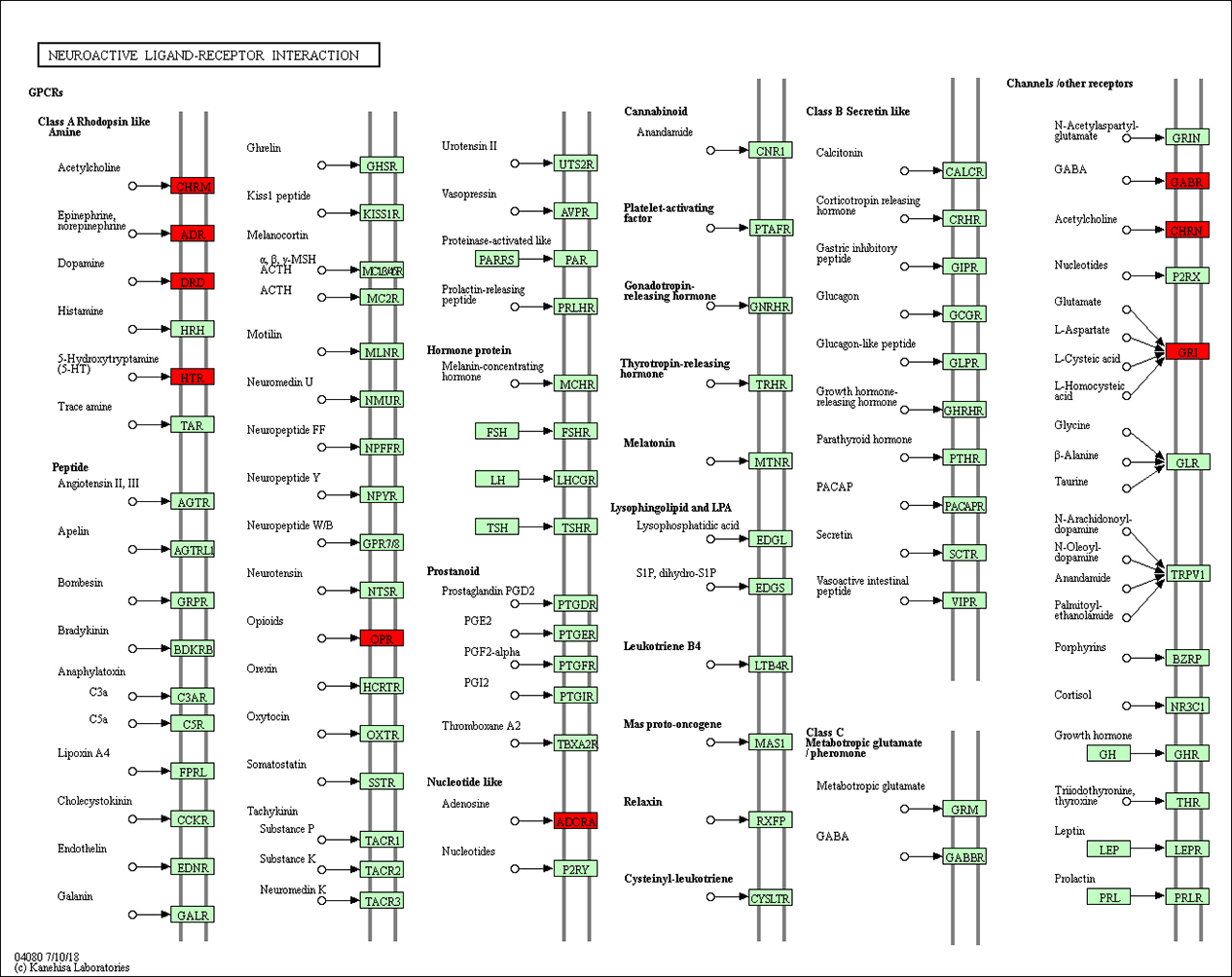

Supplement: Figure S1 — Distribution of partial targets of XYS on the Neuroactive ligand-receptor interaction signaling pathway. The red nodes are potential targets. The green nodes are relevant targets in the pathway. [file Image_1.JPEG]
