# Supplementary material for: An Integrated Pharmacology-Based Analysis for Antidepressant Mechanism of Chinese Herbal Formula Xiao-Yao-San
Source: Front Pharmacol. 2020 Mar 18;11:284. doi: 10.3389/fphar.2020.00284 (PMC7094752; doi:10.3389/fphar.2020.00284)
Supplement: Table S1 — Bioactive ingredients of XYS. [file Table_1.docx]

**Table S1. Bioactive ingredients of XYS**

| **No.** | **Compound Name** | **MW** | **AlogP** | **Hdon** | **Hacc** | **OB (%)** | **DL** | **Herb** |
| --- | --- | --- | --- | --- | --- | --- | --- | --- |
| C01 | (-)-Medicocarpin | 432.46 | 0.75 | 4 | 9 | 40.99 | 0.95 | GRH |
| C02 | (+)-Anomalin | 426.5 | 5.05 | 0 | 7 | 46.06 | 0.66 | BR |
| C03 | (+)-catechin | 290.29 | 1.92 | 5 | 6 | 54.83 | 0.24 | PN |
| C04 | (2R)-2-[(5R,10S,13R,14R,16R,17R)-16-hydroxy-3-keto-4,4,10,13,14-pentamethyl-1,2,5,6,12,15,16,17-octahydrocyclopenta[a]phenanthren-17-yl]-5-isopropyl-hex-5-enoic acid | 482.77 | 5.68 | 2 | 4 | 38.26 | 0.82 | PR |
| C05 | (2R)-2-[(3S,5R,10S,13R,14R,16R,17R)-3,16-dihydroxy-4,4,10,13,14-pentamethyl-2,3,5,6,12,15,16,17-octahydro-1H-cyclopenta[a]phenanthren-17-yl]-6-methylhept-5-enoic acid | 470.76 | 5.41 | 3 | 4 | 30.93 | 0.81 | PR |
| C06 | (2R)-7-hydroxy-2-(4-hydroxyphenyl)chroman-4-one | 256.27 | 2.57 | 2 | 4 | 71.12 | 0.18 | GRH |
| C07 | (2S)-2-[4-hydroxy-3-(3-methylbut-2-enyl)phenyl]-8,8-dimethyl-2,3-dihydropyrano[2,3-f]chromen-4-one | 390.51 | 5.48 | 1 | 4 | 31.79 | 0.72 | GRH |
| C08 | (2S)-6-(2,4-dihydroxyphenyl)-2-(2-hydroxypropan-2-yl)-4-methoxy-2,3-dihydrofuro[3,2-g]chromen-7-one | 384.41 | 2.96 | 3 | 7 | 60.25 | 0.63 | GRH |
| C09 | (2S)-7-hydroxy-2-(4-hydroxyphenyl)-8-(3-methylbut-2-enyl)chroman-4-one | 324.4 | 4.42 | 2 | 4 | 36.57 | 0.32 | GRH |
| C10 | (3S,5R,8R,9R,10S,14S)-3,17-dihydroxy-4,4,8,10,14-pentamethyl-2,3,5,6,7,9-hexahydro-1H-cyclopenta[a]phenanthrene-15,16-dione | 358.52 | 2.69 | 2 | 4 | 43.56 | 0.53 | PN |
| C11 | (E)-1-(2,4-dihydroxyphenyl)-3-(2,2-dimethylchromen-6-yl)prop-2-en-1-one | 322.38 | 3.96 | 2 | 4 | 39.62 | 0.35 | GRH |
| C12 | (E)-3-[3,4-dihydroxy-5-(3-methylbut-2-enyl)phenyl]-1-(2,4-dihydroxyphenyl)prop-2-en-1-one | 340.4 | 4.49 | 4 | 5 | 46.27 | 0.31 | GRH |
| C13 | 1,3-dihydroxy-8,9-dimethoxy-6-benzofurano[3,2-c]chromenone | 328.29 | 2.98 | 2 | 7 | 62.9 | 0.53 | GRH |
| C14 | 1,3-dihydroxy-9-methoxy-6-benzofurano[3,2-c]chromenone | 298.26 | 2.99 | 2 | 6 | 48.14 | 0.43 | GRH |
| C15 | 14-acetyl-12-senecioyl-2E,8Z,10E-atractylentriol | 356.45 | 3.54 | 1 | 5 | 63.37 | 0.3 | RAM |
| C16 | 18α-hydroxyglycyrrhetic acid | 486.76 | 4.55 | 3 | 5 | 41.16 | 0.71 | GRH |
| C17 | 1-Methoxyphaseollidin | 354.43 | 4.25 | 2 | 5 | 69.98 | 0.64 | GRH |
| C18 | 2-(3,4-dihydroxyphenyl)-5,7-dihydroxy-6-(3-methylbut-2-enyl)chromone | 354.38 | 3.92 | 4 | 6 | 44.15 | 0.41 | GRH |
| C19 | 2-[(3R)-8,8-dimethyl-3,4-dihydro-2H-pyrano[6,5-f]chromen-3-yl]-5-methoxyphenol | 338.43 | 4.2 | 1 | 4 | 36.21 | 0.52 | GRH |
| C20 | 3-(2,4-dihydroxyphenyl)-8-(1,1-dimethylprop-2-enyl)-7-hydroxy-5-methoxy-coumarin | 368.41 | 4.03 | 3 | 6 | 59.62 | 0.43 | GRH |
| C21 | 3-(3,4-dihydroxyphenyl)-5,7-dihydroxy-8-(3-methylbut-2-enyl)chromone | 354.38 | 3.65 | 4 | 6 | 66.37 | 0.41 | GRH |
| C22 | 3'-Hydroxy-4'-O-Methylglabridin | 354.43 | 3.93 | 2 | 5 | 43.71 | 0.57 | GRH |
| C23 | 3'-Methoxyglabridin | 354.43 | 3.93 | 2 | 5 | 46.16 | 0.57 | GRH |
| C24 | 3β-acetoxyatractylone | 274.39 | 3.39 | 0 | 3 | 54.07 | 0.22 | RAM |
| C25 | 5,7-dihydroxy-3-(4-methoxyphenyl)-8-(3-methylbut-2-enyl)chromone | 352.41 | 4.17 | 2 | 5 | 30.49 | 0.41 | GRH |
| C26 | 6-methylgingediacetate2 | 394.56 | 4.55 | 0 | 6 | 48.73 | 0.32 | RZR |
| C27 | 6-prenylated eriodictyol | 356.4 | 3.89 | 4 | 6 | 39.22 | 0.41 | GRH |
| C28 | 7,2',4'-trihydroxy－5-methoxy-3－arylcoumarin | 300.28 | 2.56 | 3 | 6 | 83.71 | 0.27 | GRH |
| C29 | 7-Acetoxy-2-methylisoflavone | 294.32 | 3.15 | 0 | 4 | 38.92 | 0.26 | GRH |
| C30 | 7-Methoxy-2-methyl isoflavone | 266.31 | 3.36 | 0 | 3 | 42.56 | 0.2 | GRH |
| C31 | 8-(6-hydroxy-2-benzofuranyl)-2,2-dimethyl-5-chromenol | 308.35 | 4.2 | 2 | 4 | 58.44 | 0.38 | GRH |
| C32 | 8-prenylated eriodictyol | 356.4 | 3.89 | 4 | 6 | 53.79 | 0.4 | GRH |
| C33 | 8β-ethoxy atractylenolide Ⅲ | 276.41 | 3.68 | 0 | 3 | 35.95 | 0.21 | RAM |
| C34 | acacetin | 284.28 | 2.59 | 2 | 5 | 34.97 | 0.24 | MH |
| C35 | aloe-emodin | 270.25 | 1.67 | 3 | 5 | 83.38 | 0.24 | MH |
| C36 | Areapillin | 360.34 | 2.29 | 3 | 8 | 48.96 | 0.41 | BR |
| C37 | Baicalin | 446.39 | 0.64 | 6 | 11 | 40.12 | 0.75 | BR |
| C38 | beta-sitosterol | 414.79 | 8.08 | 1 | 1 | 36.91 | 0.75 | AS/PN/ZRR |
| C39 | Calycosin | 284.28 | 2.32 | 2 | 5 | 47.75 | 0.24 | GRH |
| C40 | Cerevisterol | 430.74 | 5.15 | 3 | 3 | 37.96 | 0.77 | PR |
| C41 | Cubebin | 356.4 | 3.19 | 1 | 6 | 57.13 | 0.64 | BR |
| C42 | dehydroglyasperins C | 340.4 | 4.3 | 4 | 5 | 53.82 | 0.37 | GRH |
| C43 | DFV | 256.27 | 2.57 | 2 | 4 | 32.76 | 0.18 | GRH |
| C44 | Diosmetin | 300.28 | 2.32 | 3 | 6 | 31.14 | 0.27 | MH |
| C45 | Semilicoisoflavone B | 352.36 | 2.85 | 3 | 6 | 48.78 | 0.55 | GRH |
| C46 | ergosta-7,22E-dien-3beta-ol | 398.74 | 7.18 | 1 | 1 | 43.51 | 0.72 | PR |
| C47 | Ergosterol peroxide | 430.74 | 7.17 | 1 | 3 | 40.36 | 0.81 | PR |
| C48 | eriodictyol | 288.27 | 2.03 | 4 | 6 | 71.79 | 0.24 | MH |
| C49 | euchrenone | 406.56 | 6.35 | 1 | 4 | 30.29 | 0.57 | GRH |
| C50 | Eurycarpin A | 338.38 | 3.92 | 3 | 5 | 43.28 | 0.37 | GRH |
| C51 | formononetin | 268.28 | 2.58 | 1 | 4 | 69.67 | 0.21 | GRH |
| C52 | gadelaidic acid | 310.58 | 7.75 | 1 | 2 | 30.7 | 0.2 | GRH |
| C53 | Gancaonin A | 352.41 | 4.17 | 2 | 5 | 51.08 | 0.4 | GRH |
| C54 | Gancaonin B | 368.41 | 3.91 | 3 | 6 | 48.79 | 0.45 | GRH |
| C55 | Gancaonin G | 352.41 | 4.17 | 2 | 5 | 60.44 | 0.39 | GRH |
| C56 | Gancaonin H | 420.49 | 4.71 | 3 | 6 | 50.1 | 0.78 | GRH |
| C57 | Genkwanin | 284.28 | 2.59 | 2 | 5 | 37.13 | 0.24 | MH |
| C58 | Glabranin | 324.4 | 4.42 | 2 | 4 | 52.9 | 0.31 | GRH |
| C59 | Glabrene | 322.38 | 3.77 | 2 | 4 | 46.27 | 0.44 | GRH |
| C60 | Glabridin | 324.4 | 3.95 | 2 | 4 | 53.25 | 0.47 | GRH |
| C61 | Glabrone | 336.36 | 3.12 | 2 | 5 | 52.51 | 0.5 | GRH |
| C62 | Glepidotin A | 338.38 | 3.9 | 3 | 5 | 44.72 | 0.35 | GRH |
| C63 | Glepidotin B | 340.4 | 3.88 | 3 | 5 | 64.46 | 0.34 | GRH |
| C64 | glyasperin B | 370.43 | 4.02 | 3 | 6 | 65.22 | 0.44 | GRH |
| C65 | Glyasperin C | 356.45 | 4.73 | 3 | 5 | 45.56 | 0.4 | GRH |
| C66 | glyasperin F | 354.38 | 2.97 | 3 | 6 | 75.84 | 0.54 | GRH |
| C67 | Glyasperins M | 368.41 | 3.22 | 2 | 6 | 72.67 | 0.59 | GRH |
| C68 | Glycyrin | 382.44 | 4.67 | 2 | 6 | 52.61 | 0.47 | GRH |
| C69 | Glycyrol | 366.39 | 4.85 | 2 | 6 | 90.78 | 0.67 | GRH |
| C70 | glycyroside | 562.57 | -0.73 | 6 | 13 | 37.25 | 0.79 | GRH |
| C71 | Glypallichalcone | 284.33 | 3.4 | 1 | 4 | 61.6 | 0.19 | GRH |
| C72 | Glyzaglabrin | 298.26 | 2.1 | 2 | 6 | 61.07 | 0.35 | GRH |
| C73 | hederagenin | 414.79 | 8.08 | 1 | 1 | 36.91 | 0.75 | PR |
| C74 | HMO | 268.28 | 2.58 | 1 | 4 | 38.37 | 0.21 | GRH |
| C75 | Inermine | 284.28 | 2.44 | 1 | 5 | 75.18 | 0.54 | GRH |
| C76 | Inflacoumarin A | 322.38 | 4.7 | 2 | 4 | 39.71 | 0.33 | GRH |
| C77 | Isoglycyrol | 366.39 | 4.36 | 1 | 6 | 44.7 | 0.84 | GRH |
| C78 | Isolicoflavonol | 354.38 | 3.63 | 4 | 6 | 45.17 | 0.42 | GRH |
| C79 | isorhamnetin | 316.28 | 1.76 | 4 | 7 | 49.6 | 0.31 | BR/ GRH |
| C80 | Isotrifoliol | 298.26 | 2.99 | 2 | 6 | 31.94 | 0.42 | GRH |
| C81 | Jaranol | 314.31 | 2.09 | 2 | 6 | 50.83 | 0.29 | GRH |
| C82 | kaempferol | 286.25 | 1.77 | 4 | 6 | 41.88 | 0.24 | BR/ GRH /PN |
| C83 | Kanzonol F | 420.54 | 5.3 | 1 | 5 | 32.47 | 0.89 | GRH |
| C84 | kanzonols W | 336.36 | 3.63 | 2 | 5 | 50.48 | 0.52 | GRH |
| C85 | Licoagrocarpin | 338.43 | 4.51 | 1 | 4 | 58.81 | 0.58 | GRH |
| C86 | Licoagroisoflavone | 336.36 | 3.48 | 2 | 5 | 57.28 | 0.49 | GRH |
| C87 | licochalcone a | 338.43 | 4.62 | 2 | 4 | 40.79 | 0.29 | GRH |
| C88 | Licochalcone B | 286.3 | 2.88 | 3 | 5 | 76.76 | 0.19 | GRH |
| C89 | licochalcone G | 354.43 | 4.35 | 3 | 5 | 49.25 | 0.32 | GRH |
| C90 | Licocoumarone | 340.4 | 4.98 | 3 | 5 | 33.21 | 0.36 | GRH |
| C91 | licoisoflavanone | 354.38 | 2.97 | 3 | 6 | 52.47 | 0.54 | GRH |
| C92 | Licoisoflavone | 354.38 | 3.65 | 4 | 6 | 41.61 | 0.42 | GRH |
| C93 | Licoisoflavone B | 352.36 | 2.85 | 3 | 6 | 38.93 | 0.55 | GRH |
| C94 | licopyranocoumarin | 384.41 | 3.04 | 3 | 7 | 80.36 | 0.65 | GRH |
| C95 | licorice glycoside E | 693.71 | 1.59 | 7 | 14 | 32.89 | 0.27 | GRH |
| C96 | Licoricone | 382.44 | 4.16 | 2 | 6 | 63.58 | 0.47 | GRH |
| C97 | Linarin | 592.6 | -0.18 | 7 | 14 | 39.84 | 0.71 | MH |
| C98 | Linoleyl acetate | 308.56 | 6.85 | 0 | 2 | 42.1 | 0.2 | BR |
| C99 | liquiritin | 418.43 | 0.66 | 5 | 9 | 65.69 | 0.74 | GRH |
| C100 | Longikaurin A | 348.48 | 1.16 | 3 | 5 | 47.72 | 0.53 | BR |
| C101 | Lupiwighteone | 338.38 | 3.92 | 3 | 5 | 51.64 | 0.37 | GRH |
| C102 | luteolin | 286.25 | 2.07 | 4 | 6 | 36.16 | 0.25 | MH |
| C103 | Glycyrrhiza flavonol A | 370.38 | 2.17 | 4 | 7 | 41.28 | 0.6 | GRH |
| C104 | Mairin | 456.78 | 6.52 | 2 | 3 | 55.38 | 0.78 | GRH /PN |
| C105 | Medicarpin | 270.3 | 2.66 | 1 | 4 | 49.22 | 0.34 | GRH |
| C106 | naringenin | 272.27 | 2.3 | 3 | 5 | 59.29 | 0.21 | MH/ GRH |
| C107 | Odoratin | 314.31 | 2.3 | 2 | 6 | 49.95 | 0.3 | GRH |
| C108 | paeoniflorgenone | 318.35 | 0.79 | 1 | 6 | 87.59 | 0.37 | PN |
| C109 | paeoniflorin | 480.51 | -1.28 | 5 | 11 | 53.87 | 0.79 | PN |
| C110 | petunidin | 317.29 | 1.65 | 5 | 7 | 30.05 | 0.31 | BR |
| C111 | Phaseol | 336.36 | 4.87 | 2 | 5 | 78.77 | 0.58 | GRH |
| C112 | Phaseolinisoflavan | 324.4 | 3.95 | 2 | 4 | 32.01 | 0.45 | GRH |
| C113 | quercetin | 302.25 | 1.5 | 5 | 7 | 46.43 | 0.28 | BR/ GRH |
| C114 | Quercetin der. | 330.31 | 1.82 | 3 | 7 | 46.45 | 0.33 | GRH |
| C115 | shinpterocarpin | 322.38 | 3.46 | 1 | 4 | 80.3 | 0.73 | GRH |
| C116 | Sigmoidin-B | 356.4 | 3.89 | 4 | 6 | 34.88 | 0.41 | GRH |
| C117 | Saikosaponin D | 781.1 | 1.11 | 8 | 13 | 34.39 | 0.09 | BR |
| C118 | Stigmasterol | 412.77 | 7.64 | 1 | 1 | 43.83 | 0.76 | AS/BR/ZRR |
| C119 | trametenolic acid | 456.78 | 7.03 | 2 | 3 | 38.71 | 0.8 | PR |
| C120 | Vestitol | 272.32 | 3.15 | 2 | 4 | 74.66 | 0.21 | GRH |
| C121 | Xambioona | 388.49 | 4.68 | 0 | 4 | 54.85 | 0.87 | GRH |
| C122 | α-spinasterol | 412.77 | 7.64 | 1 | 1 | 42.98 | 0.76 | BR |
| C123 | (2R)-2-[(3S,5R,10S,13R,14R,16R,17R)-3,16-dihydroxy-4,4,10,13,14-pentamethyl-2,3,5,6,12,15,16,17-octahydro-1H-cyclopenta[a]phenanthren-17-yl]-5-isopropyl-hex-5-enoic acid | 484.79 | 5.72 | 3 | 4 | 31.07 | 0.82 | PR |
| C124 | (3S,8S,9S,10R,13R,14S,17R)-10,13-dimethyl-17-[(2R,5S)-5-propan-2-yloctan-2-yl]-2,3,4,7,8,9,11,12,14,15,16,17-dodecahydro-1H-cyclopenta[a]phenanthren-3-ol | 428.82 | 8.54 | 1 | 1 | 36.23 | 0.78 | AMR |
| C125 | 11alpha,12alpha-epoxy-3beta-23-dihydroxy-30-norolean-20-en-28,12beta-olide | 470.71 | 3.91 | 2 | 5 | 64.77 | 0.38 | PN |
| C126 | 12-senecioyl-2E,8E,10E-atractylentriol | 312.39 | 2.5 | 0 | 4 | 62.4 | 0.22 | AMR |
| C127 | 14-acetyl-12-senecioyl-2E,8E,10E-atractylentriol | 355.44 | 3.21 | 0 | 5 | 60.31 | 0.31 | AMR |
| C128 | 3,22-Dihydroxy-11-oxo-delta(12)-oleanene-27-alpha-methoxycarbonyl-29-oic acid | 512.75 | 4.37 | 1 | 6 | 34.32 | 0.55 | GRH |
| C129 | 3,5,6,7-tetramethoxy-2-(3,4,5-trimethoxyphenyl)chromone | 432.46 | 2.54 | 0 | 9 | 31.97 | 0.59 | BR |
| C130 | 3beta-Hydroxy-24-methylene-8-lanostene-21-oic acid | 470.81 | 7.33 | 2 | 3 | 38.7 | 0.81 | PR |
| C131 | 7,9(11)-dehydropachymic acid | 526.83 | 6.1 | 2 | 5 | 35.11 | 0.81 | PR |
| C132 | albiflorin_qt | 318.35 | 0.42 | 2 | 6 | 66.64 | 0.33 | PN |
| C133 | benzoyl paeoniflorin | 584.62 | 0.81 | 4 | 12 | 31.27 | 0.75 | PN |
| C134 | dehydroeburicoic acid | 453.75 | 6.35 | 1 | 3 | 44.17 | 0.83 | PR |
| C135 | Dihydrocapsaicin | 307.48 | 4.33 | 2 | 4 | 47.07 | 0.19 | ZRR |
| C136 | Fortunellin | 592.6 | -0.18 | 7 | 14 | 35.65 | 0.74 | MH |
| C137 | icos-5-enoic acid | 310.58 | 7.75 | 1 | 2 | 30.7 | 0.2 | GRH |
| C138 | Lactiflorin | 462.49 | -0.57 | 3 | 10 | 49.12 | 0.8 | PN |
| C139 | Octalupine | 264.41 | -0.07 | 1 | 4 | 47.82 | 0.28 | BR |
| C140 | pachymic acid | 528.85 | 6.54 | 2 | 5 | 33.63 | 0.81 | PR |
| C141 | paeoniflorin_qt | 318.35 | 0.46 | 2 | 6 | 68.18 | 0.4 | PN |
| C142 | Poricoic acid A | 498.77 | 5.94 | 3 | 5 | 30.61 | 0.76 | PR |
| C143 | Poricoic acid B | 484.74 | 5.64 | 3 | 5 | 30.52 | 0.75 | PR |
| C144 | poricoic acid C | 482.77 | 7.11 | 2 | 4 | 38.15 | 0.75 | PR |
| C145 | poriferast-5-en-3beta-ol | 414.79 | 8.08 | 1 | 1 | 36.91 | 0.75 | ZRR |
| C146 | saikosaponin c_qt | 472.78 | 3.71 | 3 | 4 | 30.5 | 0.63 | BR |
| C147 | Sainfuran | 286.3 | 3.38 | 2 | 5 | 79.91 | 0.23 | BR |
| C148 | Troxerutin | 346.56 | 5.89 | 3 | 3 | 31.6 | 0.28 | BR |
| C149 | α-Amyrin | 426.8 | 7.35 | 1 | 1 | 39.51 | 0.76 | AMR |
