# Supplementary material for: An Integrated Pharmacology-Based Analysis for Antidepressant Mechanism of Chinese Herbal Formula Xiao-Yao-San
Source: Front Pharmacol. 2020 Mar 18;11:284. doi: 10.3389/fphar.2020.00284 (PMC7094752; doi:10.3389/fphar.2020.00284)
Supplement: Table S2 — The DEmRNAs of GSE12654. [file Table_2.DOCX]

**Table S2**

| **Gene symbol** | **logFC** | **P.Value** | **Dysregulated** |
| --- | --- | --- | --- |
| ZFR | -1.5033 | 0.001974 | Down |
| LECT2 | -1.46529 | 0.000687 | Down |
| WIPF1 | -1.45022 | 0.003618 | Down |
| OR1E1 | -1.38431 | 0.000796 | Down |
| TNPO2 | -1.37161 | 0.000563 | Down |
| KIF25-AS1 | -1.35696 | 0.006029 | Down |
| ANGPT1 | -1.35415 | 0.000539 | Down |
| FBXL4 | -1.34412 | 0.015206 | Down |
| NKTR | -1.32633 | 0.011094 | Down |
| ITK | -1.31651 | 0.014646 | Down |
| FYB | -1.27747 | 0.01999 | Down |
| TNFRSF17 | -1.27729 | 0.006584 | Down |
| FLT3 | -1.26717 | 0.001604 | Down |
| FRMPD4 | -1.23146 | 0.01818 | Down |
| OLFM4 | 1.211969 | 0.031754 | Up |
| ELP4 | 1.219984 | 0.013413 | Up |
| PDE5A | 1.22132 | 0.020132 | Up |
| AURKA | 1.235354 | 0.007544 | Up |
| CYP19A1 | 1.294443 | 0.006219 | Up |
| GPR4 | 1.303884 | 0.005567 | Up |
| NF1P9 | 1.311757 | 0.034738 | Up |
| RAB9BP1 | 1.313375 | 0.006977 | Up |
| SLC14A1 | 1.359169 | 0.006519 | Up |
| ZKSCAN8 | 1.361156 | 0.017143 | Up |
| ABCC2 | 1.37396 | 0.01798 | Up |
| THOC5 | 1.394613 | 0.038608 | Up |
| ETV6 | 1.407243 | 0.000174 | Up |
| TRHR | 1.500296 | 0.010793 | Up |
| DKK1 | 1.52522 | 0.012161 | Up |
