# Supplementary material for: An Integrated Pharmacology-Based Analysis for Antidepressant Mechanism of Chinese Herbal Formula Xiao-Yao-San
Source: Front Pharmacol. 2020 Mar 18;11:284. doi: 10.3389/fphar.2020.00284 (PMC7094752; doi:10.3389/fphar.2020.00284)
Supplement: Table S3 — (A) PCA analysis for physicochemical properties of potential antidepressant molecules; (B) Correlation analysis for properties between each molecular group. [file Table_3.DOCX]

**Table S3.**

1. **PCA analysis for physicochemical properties of potential** **antidepressant molecules.**

| **Physicochemical property** | **PC1** | **PC2** | **PC3** | **PC4** |
| --- | --- | --- | --- | --- |
| **MW** | 0.9999 | -0.009 | -0.010 | 0.0012 |
| **AlogP** | 0.0025 | -0.6091 | 0.7902 | -0.068 |
| **Hdon** | 0.006 | 0.3736 | 0.3614 | 0.8543 |
| **Hacc** | 0.0119 | 0.6996 | 0.4948 | -0.515 |

1. **Correlation analysis for properties between each molecular group**

| **Property** | **r** | **P-value** |
| --- | --- | --- |
| **MW** | 0.3404 | < 0.0001 |
| **AlogP** | 0.1969 | 0.003 |
| **Hdon** | 0.3018 | < 0.0001 |
| **Hacc** | 0.351 | < 0.0001 |
