# Supplementary material for: An Integrated Pharmacology-Based Analysis for Antidepressant Mechanism of Chinese Herbal Formula Xiao-Yao-San
Source: Front Pharmacol. 2020 Mar 18;11:284. doi: 10.3389/fphar.2020.00284 (PMC7094752; doi:10.3389/fphar.2020.00284)
Supplement: Table S4 — C-DTs network. [file Table_4.DOCX]

| **No.** | **Compound** | **Degree** | **Targets** |
| --- | --- | --- | --- |
| C01 | (-)-Medicocarpin | 2 | PTGS2; ACHE |
| C02 | (+)-Anomalin | 23 | TTR; SHBG; RORA; PTGS2; PLA2G10; NR3C2; NR1H2; MIF; MAPK8; MAPK14; MAPK1; MAOB; IGF1R; HSP90AA1; HSD11B1; GBA; FGFR1; ESR1; EGFR; DPP4; AR; ANG; ALB; AKR1C3 |
| C03 | (+)-catechin | 19 | SOD2; PTGS2; PTGS1; PRKACA; PLA2G10; NR3C2; MAPK14; KDR; HSP90AA1; HSD11B1; GSR; GSK3B; ESR2; ESR1; EGFR; CAT; CALM2; AR; AKR1C3; AKR1C1 |
| C04 | α-spinasterol | 1 | NR3C2 |
| C05 | (2R)-2-[(3S,5R,10S,13R,14R,16R,17R)-3,16-dihydroxy-4,4,10,13,14-pentamethyl-2,3,5,6,12,15,16,17-octahydro-1H-cyclopenta[a]phenanthren-17-yl]-6-methylhept-5-enoic acid | 1 | NR3C2 |
| C06 | (2R)-7-hydroxy-2-(4-hydroxyphenyl)chroman-4-one | 8 | SLC6A4; PTGS2; PTGS1; PRKACA; MAOB; GABRA1; ESR1; CALM2; ADRB2 |
| C07 | (2S)-2-[4-hydroxy-3-(3-methylbut-2-enyl)phenyl]-8,8-dimethyl-2,3-dihydropyrano[2,3-f]chromen-4-one | 7 | PTGS2; NOS2; MAPK14; GSK3B; ESR2; ESR1; CALM2; AR |
| C08 | (2S)-6-(2,4-dihydroxyphenyl)-2-(2-hydroxypropan-2-yl)-4-methoxy-2,3-dihydrofuro[3,2-g]chromen-7-one | 10 | PTGS2; NOS2; MAPK14; KDR; GSK3B; ESR2; ESR1; DPP4; CALM2; AR; ACHE |
| C09 | (2S)-7-hydroxy-2-(4-hydroxyphenyl)-8-(3-methylbut-2-enyl)chroman-4-one | 6 | PTGS2; PTGS1; NOS2; ESR2; ESR1; CALM2; ADRB2 |
| C10 | (3S,5R,8R,9R,10S,14S)-3,17-dihydroxy-4,4,8,10,14-pentamethyl-2,3,5,6,7,9-hexahydro-1H-cyclopenta[a]phenanthrene-15,16-dione | 1 | NR3C2 |
| C11 | (E)-1-(2,4-dihydroxyphenyl)-3-(2,2-dimethylchromen-6-yl)prop-2-en-1-one | 9 | PTGS2; PTGS1; NOS2; MAPK14; GSK3B; ESR2; ESR1; CALM2; AR |
| C12 | (E)-3-[3,4-dihydroxy-5-(3-methylbut-2-enyl)phenyl]-1-(2,4-dihydroxyphenyl)prop-2-en-1-one | 6 | PTGS2; MAPK14; GSK3B; ESR1; CALM2; AR |
| C13 | 1,3-dihydroxy-8,9-dimethoxy-6-benzofurano[3,2-c]chromenone | 5 | PRKACA; MAPK14; GSK3B; ESR1; AR |
| C14 | 1,3-dihydroxy-9-methoxy-6-benzofurano[3,2-c]chromenone | 5 | PRKACA; MAPK14; GSK3B; ESR2; ESR1 |
| C15 | 14-acetyl-12-senecioyl-2E,8Z,10E-atractylentriol | 1 | PTGS2 |
| C16 | 18α-hydroxyglycyrrhetic acid | 18 | WAS; SHBG; RORA; REN; PLA2G2A; NR1H2; NOS3; MET; MAPK14; KDR; HSD11B1; EGFR; DPP4; CALM2; ALB; AKR1C3; AKR1C1; ADH1B |
| C17 | 1-Methoxyphaseollidin | 18 | SHBG; PTGS2; PTGS1; NOS3; NOS2; MIF; MAPK8; MAPK14; KDR; GSR; GSK3B; ESR2; ESR1; DUSP6; CALM2; AR; ANG; ADRB2 |
| C18 | 2-(3,4-dihydroxyphenyl)-5,7-dihydroxy-6-(3-methylbut-2-enyl)chromone | 5 | PTGS2; DPP4; CALM2; AR; ADRB2 |
| C19 | 2-[(3R)-8,8-dimethyl-3,4-dihydro-2H-pyrano[6,5-f]chromen-3-yl]-5-methoxyphenol | 14 | SLC6A3; PTGS2; PTGS1; PRKACA; NOS3; NOS2; MAPK14; GSK3B; ESR2; ESR1; CALM2; AR; ADRB2; ACHE |
| C20 | 3-(2,4-dihydroxyphenyl)-8-(1,1-dimethylprop-2-enyl)-7-hydroxy-5-methoxy-coumarin | 10 | PTGS2; NOS2; MAPK14; KDR; GSK3B; ESR2; ESR1; DPP4; CALM2; AR |
| C21 | 3-(3,4-dihydroxyphenyl)-5,7-dihydroxy-8-(3-methylbut-2-enyl)chromone | 7 | PTGS2; NOS2; MAPK14; GSK3B; ESR1; CALM2; AR |
| C22 | 3'-Hydroxy-4'-O-Methylglabridin | 12 | PTGS2;  PTGS1; PRKACA; NOS2; MAPK14; KDR; GSK3B; ESR2; ESR1; CALM2; AR; ADRB2 |
| C23 | 3'-Methoxyglabridin | 11 | PTGS2; PTGS1; NOS2; MAPK14; GSK3B; ESR2; ESR1; CALM2; AR; ADRB2; ACHE |
| C24 | 3β-acetoxyatractylone | 10 | PTGS2; OPRM1; NOS3; GABRA1; DPP4; CHRM2; AR; ADRB2; ADRA1A; ACHE |
| C25 | 5,7-dihydroxy-3-(4-methoxyphenyl)-8-(3-methylbut-2-enyl)chromone | 9 | PTGS2; NOS2; MAPK14; GSK3B; ESR2; ESR1; DPP4; CALM2; AR |
| C26 | 6-methylgingediacetate2 | 1 | CALM2 |
| C27 | 6-prenylated eriodictyol | 4 | PTGS2; NOS2; ESR1; CALM2 |
| C28 | 7,2',4'-trihydroxy－5-methoxy-3－arylcoumarin | 10 | PTGS2; PTGS1; PRKACA; NOS2; MAPK14; GSK3B; ESR2; ESR1; DPP4; AR |
| C29 | 7-Acetoxy-2-methylisoflavone | 23 | TTR; PTGS2; PTGS1; NR3C2; NR1H2; NOS3; NOS2; MIF; MAPK14; LTA4H; IGF1R; GSR; GSK3B; GABRA1; ESR2; ESR1; EGFR; DPP4; CALM2; AR; ANG; ADRB2; ACHE |
| C30 | 7-Methoxy-2-methyl isoflavone | 22 | SLC6A4; SLC6A3; PTGS2; PTGS1; PRKACA; OPRM1; NOS3; NOS2; MAPK14; MAOB; LTA4H; GSK3B; GABRA1; ESR2; ESR1; DRD1; DPP4; CALM2; AR; ADRB2; ADRB1; ACHE |
| C31 | 8-(6-hydroxy-2-benzofuranyl)-2,2-dimethyl-5-chromenol | 3 | PTGS2; NOS2; ESR1 |
| C32 | 8-prenylated eriodictyol | 3 | PTGS2; ESR1; CALM2 |
| C33 | 8β-ethoxy atractylenolide Ⅲ | 2 | PTGS2; GABRA1 |
| C34 | acacetin | 23 | TP53; SHBG; PTGS2; PTGS1; PRKACA; NOS3; NOS2; MIF; MAPK8; MAPK14; KDR; GSR; FASLG; ESR2; ESR1; DUSP6; DPP4; CALM2; BCL2; BAX; AR; ANG; ADRB2 |
| C35 | aloe-emodin | 23 | TP53; TNF; SOD2; PTGS2; PTGS1; PRKCE; PRKACA; NOS3; MAPK8; MAPK14; MAOB; LTA4H; IL1B; HSP90AA1; HSD11B1; GBA; FGFR1; ESR2; ESR1; EGFR; BAX; ANG; AKR1C3 |
| C36 | Areapillin | 6 | PTGS2; NOS2; ESR2; DPP4; CALM2; AR |
| C37 | Baicalin | 14 | SOD2; REN; PLA2G2A; MIF; MAPK8; IMPA2; IGF1R; GSK3B; GLO1; FGFR1; FABP5; DPP4; CYP2C9; ALB |
| C38 | beta-sitosterol | 30 | TGFB1; SLC6A4; SHBG; RORA; PTGS2; PTGS1; PRKACA; PON1; OPRM1; NR3C2; NR1H2; MAOB; HTR2A; HSD11B1; GABRA3; GABRA2; GABRA1; ESR1; EGFR; DRD1; DPP4; CHRNA2; CHRM2; CALM2; BCL2; BAX; AR; AKR1C1; ADRB2; ADRA1A |
| C39 | Calycosin | 20 | SHBG; PTGS2; PTGS1; PRKACA; PAH; NOS2; MIF; MAPK14; LTA4H; HSP90AA1; HSD11B1; GSK3B; ESR2; ESR1; DUSP6; DPP4; CALM2; AR; ANG; ADRB2 |
| C40 | Cerevisterol | 17 | WAS; SHBG; RORA; REN; NR3C2; MET; MAPK1; MAOB; HSD11B1; GSK3B; ESR1; ERBB4; EGFR; DPP4; CALM2; ALB; AKR1C1 |
| C41 | Cubebin | 11 | SOD2; SHBG; REN; PTGS2; PTGS1; PAH; MAPK14; HSD11B1; GSK3B; ESR2; ALB |
| C42 | dehydroglyasperins C | 8 | PTGS2; NOS2; MAPK14; ESR2; ESR1; CALM2; AR; ADRB2 |
| C43 | DFV | 7 | SLC6A4; PTGS2; PTGS1; PRKACA; MAOB; ESR1; ADRB2 |
| C44 | Diosmetin | 17 | SHBG; PTGS2; PTGS1; PRKACA; PAH; NR3C2; NOS3; NOS2; GSR; FGFR1; ESR2; ESR1; DUSP6; DPP4; CALM2; AR; ANG |
| C45 | Semilicoisoflavone B | 21 | SHBG; PTGS2; PAH; NOS2; MIF; MAPK14; MAOB; LTA4H; KDR; IGF1R; HSP90AA1; HSD11B1; GSK3B; FGFR1; ESR2; ESR1; EGFR; CALM2; AR; ANG; ACHE |
| C46 | ergosta-7,22E-dien-3beta-ol | 13 | WAS; SHBG; RORA; NR3C2; NR1H2; MAPK14; MAOB; HSD11B1; ERBB4; DPP4; CALM2; ALB; AKR1C1 |
| C47 | Ergosterol peroxide | 21 | WAS; TTR; SHBG; RORA; REN; PLA2G10; NR3C2; NR1H2; MET; MAPK14; KDR; IGF1R; HSP90AA1; HSD11B1; GSK3B; FABP7; ESR1; ERBB4; DPP4; CALM2; AKR1C3; AKR1C1 |
| C48 | eriodictyol | 3 | PTGS2; PTGS1; PRKACA |
| C49 | euchrenone | 15 | SHBG; PTGS2; PAH; NOS3; NOS2; MIF; HSP90AA1; HSD11B1; GSR; FGFR1; ESR2; ESR1; CALM2; AR; ANG |
| C50 | Eurycarpin A | 18 | REN; PTGS2; NOS2; MAPK8; MAPK14; MAOB; IMPA2; HSD11B1; GSK3B; GBA; ESR2; ESR1; EGFR; DPP4; CYP2C9; CALM2; AR; AKR1C1 |
| C51 | formononetin | 23 | SLC6A4; SLC6A3; SIRT1; SHBG; PTGS2; PTGS1; PRKACA; NOS3; NOS2; MAPK14; MAOB; IL4; GSK3B; ESR2; ESR1; DUSP6; DPP4; CALM2; AR; ANG; ADRB2; ADRA1A; ACHE |
| C52 | gadelaidic acid | 16 | TTR; SOD2; REN; PAH; NR3C2; MAPK14; HSP90AA1; GSR; GSK3B; ESR2; ESR1; EGFR; DPP4; AR; ANG; AKT1 |
| C53 | Gancaonin A | 22 | PTGS2; PLA2G10; NR1H2; NOS2; MET; MAPK8; KDR; HSD11B1; GSR; GSK3B; ESR2; ESR1; ERBB4; EGFR; DPP4; CALM2; AR; ANG; ALB; AKR1C3; AKR1C1; ACHE |
| C54 | Gancaonin B | 17 | SHBG; PTGS2; NOS3; NOS2; MET; KDR; HSD11B1; GSK3B; ESR2; ESR1; DUSP6; DPP4; CYP2C9; CALM2; AR; AKR1C3; ADRB2 |
| C55 | Gancaonin G | 19 | WAS; RORA; PTGS2; NR3C2; NOS3; NOS2; MET; MAPK14; MAPK1; MAOB; HSD11B1; GSK3B; FABP5; ESR2; ESR1; DPP4; CALM2; AR; AKR1C3 |
| C56 | Gancaonin H | 16 | SHBG; PTGS2; NOS3; MET; MAPK14; KDR; HSD11B1; GSK3B; ESR2; ESR1; EGFR; DUSP6; CYP2C9; CALM2; AR; AKR1C3 |
| C57 | Genkwanin | 19 | SHBG; REN; PTGS2; PTGS1; PRKACA; NR3C2; NOS2; MAPK8; MAPK14; MAPK1; MAOB; HSD11B1; ESR2; ESR1; EGFR; DPP4; CALM2; AR; ALB |
| C58 | Glabranin | 20 | TTR; RORA; PTGS2; PTGS1; PRKACA; NOS3; NOS2; MET; MAPK1; HSP90AA1; HSD11B1; GSR; FGFR1; ESR2; ESR1; EGFR; DUSP6; CALM2; AKR1C3; ADORA2A |
| C59 | Glabrene | 18 | RORA; PTGS2; PTGS1; PLA2G10; NOS2; MIF; MAPK8; MAPK14; MAOB; GSK3B; FGFR1; ESR2; ESR1; CALM2; AR; ALB; ADRB2; ADH1B |
| C60 | Glabridin | 20 | PTGS2; PRKACA; NOS2; MIF; MAPK14; MAOB; KDR; HSD11B1; GSK3B; FGFR1; ESR2; ESR1; EGFR; DUSP6; CALM2; AR; ANG; ADRB2; ADORA2A; ACHE |
| C61 | Glabrone | 25 | PTGS2; PTGS1; PLA2G10; NOS3; NOS2; MET; MAPK14; MAPK1; LTA4H; HSP90AA1; HSD11B1; GSK3B; FGFR1; ESR2; ESR1; ERBB4; EGFR; DPP4; CYP2C9; CALM2; AR; ANG; ALB; ADORA2A; ACHE |
| C62 | Glepidotin A | 19 | SHBG; PTGS2; PTGS1; NOS3; NOS2; MAPK8; MAPK14; MAPK1; MAOB; KDR; GSK3B; ESR2; ESR1; EGFR; DPP4; CYP2C9; CALM2; AR; ANG |
| C63 | Glepidotin B | 12 | SHBG; PTGS2; PTGS1; NOS3; MET; HSD11B1; ESR2; ESR1; DUSP6; CYP2C9; CALM2; AKR1C3 |
| C64 | glyasperin B | 23 | PTGS2; NR1H2; NOS3; NOS2; MIF; MET; MAPK8; MAPK14; KDR; IMPA2; HSD11B1; GSR; GSK3B; GBA; ESR2; ESR1; DPP4; CYP2C9; CALM2; AR; AKR1C3; AKR1C1; ACHE |
| C65 | Glyasperin C | 24 | TTR; RORA; REN; PTGS2; PLA2G10; NOS2; MET; MAPK14; MAPK1; MAOB; HSP90AA1; HSD11B1; GSK3B; FGFR1; ESR2; ESR1; ERBB4; DPP4; CYP2C9; CALM2; AR; ALB; AKT1; ACHE |
| C66 | glyasperin F | 18 | TTR; SOD2; REN; PTGS2; PTGS1; PAH; NOS2; MAPK14; HSP90AA1; GSR; GSK3B; ESR2; ESR1; ERBB4; CALM2; AR; ANG; AKR1C3 |
| C67 | Glyasperins M | 11 | PTGS2; PTGS1; PRKACA; NOS2; KDR; GSK3B; ESR2; ESR1; CALM2; AR; ACHE |
| C68 | Glycyrin | 23 | TTR; SOD2; REN; PTGS2; NR3C2; NOS2; MET; MAPK8; MAPK14; MAOB; KDR; HSP90AA1; HSD11B1; GSR; ESR2; ESR1; ERBB4; EGFR; DPP4; CALM2; AR; ALDH2; AKR1C3 |
| C69 | Glycyrol | 16 | WAS; REN; PTGS2; PLA2G10; NR3C2; NOS2; MET; MAPK14; KDR; HSD11B1; GSK3B; ESR2; ESR1; DUSP6; AR; AKR1C1 |
| C70 | glycyroside | 12 | TTR; PAH; IMPA2; IGF1R; HSP90AA1; GBA; ESR2; ESR1; EGFR; BAG1; ANG; ALDH2 |
| C71 | Glypallichalcone | 18 | SLC6A4; SLC6A3; PTGS2; PTGS1; PRKACA; NOS2; MIF; MAPK14; MAOB; LTA4H; HSP90AA1; GSK3B; ESR2; ESR1; EGFR; CALM2; AR; ADRB2 |
| C72 | Glyzaglabrin | 19 | PTGS2; PTGS1; PRKACA; PAH; NOS2; MIF; MAPK14; MAOB; HSD11B1; GSR; GSK3B; ESR2; ESR1; EGFR; DUSP6; DPP4; CYP2C9; AR; ANG |
| C73 | hederagenin | 26 | SOD2; SLC6A2; SHBG; RORA; REN; PTGS2; PTGS1; NR3C2; NR1H2; NOS3; MET; MAPK8; MAOB; KDR; HSD11B1; GRIA2; GABRA6; GABRA3; GABRA2; GABRA1; ESR1; EGFR; DPP4; CHRM2; ALB; ADH1B |
| C74 | HMO | 15 | SLC6A4; SLC6A3; PTGS2; PTGS1; PRKACA; NOS2; MAPK14; MAOB; GSK3B; ESR2; ESR1; DPP4; CALM2; AR; ADRB2 |
| C75 | Inermine | 19 | PTGS2; PTGS1; PRKACA; OPRM1; NR1H2; NOS3; MAPK14; HTR3A; HSD11B1; GSR; ESR1; DUSP6; DPP4; CALM2; AR; ALB; AKR1C3; AKR1C1; ADRB2 |
| C76 | Inflacoumarin A | 7 | PTGS2; PTGS1; ESR1; DPP4; CALM2; AR; ADRB2 |
| C77 | Isoglycyrol | 24 | TTR; SHBG; RORA; PTGS2; PLA2G10; NR3C2; NOS3; NOS2; MIF; MAPK8; MAPK14; MAOB; HSP90AA1; HSD11B1; GSR; GSK3B; GBA; ESR2; ESR1; EGFR; DUSP6; DPP4; AR; ALB |
| C78 | Isolicoflavonol | 12 | SOD2; PTGS2; NOS2; MAPK14; HSD11B1; GSR; GSK3B; ESR2; ESR1; ERBB4; CALM2; AR |
| C79 | isorhamnetin | 23 | SHBG; PTGS2; PTGS1; PRKACA; PAH; NOS3; NOS2; MAPK14; MAOB; GSR; GSK3B; GRIA2; GBA; GABRA1; FGFR1; ESR2; ESR1; EGFR; DPP4; CALM2; AR; ANG; ACHE |
| C80 | Isotrifoliol | 18 | TTR; SHBG; PTGS2; PRKACA; NOS2; MIF; MAPK8; MAPK14; HSD11B1; GSR; GSK3B; GBA; ESR2; ESR1; EGFR; DUSP6; AR; ALB |
| C81 | Jaranol | 16 | SHBG; PTGS2; PTGS1; NOS3; NOS2; MIF; MAPK8; MAPK14; KDR; GSR; ESR2; ESR1; DPP4; CALM2; AR; ANG |
| C82 | kaempferol | 30 | TNF; SLC6A2; SHBG; PTGS2; PTGS1; PRKACA; NOS3; NOS2; MAPK8; GSTM1; GSR; GBA; GABRA2; GABRA1; ESR2; ESR1; EGFR; DPP4; CYP1A2; CYP1A1; CHRM2; CALM2; BCL2; BAX; AR; ANG; AKT1; AKR1C3; AHSA1; ACHE |
| C83 | Kanzonol F | 19 | SHBG; PTGS2; PLA2G10; MIF; MAPK8; MAPK14; MAPK1; KDR; HSP90AA1; HSD11B1; GSK3B; ESR2; ESR1; EGFR; DUSP6; CALM2; AR; ALB; AKR1C3 |
| C84 | kanzonols W | 21 | TTR; SHBG; RORA; PTGS2; PTGS1; PLA2G10; NOS2; MAPK8; MAPK14; KDR; HSP90AA1; GSR; GSK3B; FGFR1; ESR2; ESR1; DPP4; CALM2; AR; ALB; ADH1B |
| C85 | Licoagrocarpin | 21 | WAS; SHBG; PTGS2; PTGS1; PLA2G10; NR3C2; NR1H2; NOS3; NOS2; MAPK14; MAPK1; GSK3B; FGFR2; ESR2; ESR1; CALM2; AR; ALB; AKR1C3; ADRB2; ACHE |
| C86 | Licoagroisoflavone | 9 | PTGS2; NOS2; MAPK14; GSK3B; ESR2; ESR1; DPP4; CALM2; AR |
| C87 | licochalcone a | 14 | STAT3; SLC6A3; PTGS2; PTGS1; NOS2; MAPK14; MAPK1; GSK3B; ESR2; ESR1; CALM2; BCL2; AR; ADRB2 |
| C88 | Licochalcone B | 11 | PTGS2; PTGS1; PRKACA; NOS2; MAPK14; GSK3B; ESR2; ESR1; CALM2; AR; ADRB2 |
| C89 | licochalcone G | 9 | PTGS2; NOS2; MAPK14; KDR; GSK3B; ESR2; ESR1; CALM2; AR |
| C90 | Licocoumarone | 14 | SOD2; SHBG; PLA2G10; NR3C2; MET; HSP90AA1; HSD11B1; GSK3B; ESR2; ESR1; ERBB4; CYP2C9; AR; AKR1C1 |
| C91 | licoisoflavanone | 21 | REN; PTGS2; PTGS1; NR3C2; NOS2; MIF; MET; MAPK8; MAOB; LTA4H; HSD11B1; GSR; GSK3B; GBA; ESR2; ESR1; EGFR; CALM2; AR; AKR1C1; ACHE |
| C92 | Licoisoflavone | 8 | PTGS2; NOS2; MAPK14; KDR; ESR1; DPP4; CALM2; AR |
| C93 | Licoisoflavone B | 16 | PTGS2; NOS2; MIF; MAPK14; MAOB; KDR; HSD11B1; GSK3B; FGFR1; ESR2; ESR1; EGFR; CALM2; AR; ADORA2A; ACHE |
| C94 | licopyranocoumarin | 23 | TTR; SOD2; SHBG; PTGS2; PLA2G10; NR3C2; NOS2; MIF; MAPK8; MAPK14; MAOB; KDR; HSP90AA1; HSD11B1; GSR; GBA; ESR2; ESR1; EGFR; CALM2; AR; ADH1B; ACHE |
| C95 | licorice glycoside E | 9 | TTR; PAH; HSP90AA1; HSD11B1; GSR; GSK3B; GBA; ANG; ALDH2 |
| C96 | Licoricone | 17 | TTR; PTGS2; NR3C2; NOS2; MAPK8; MAPK14; KDR; HSP90AA1; HSD11B1; GSK3B; GBA; ESR2; ESR1; ERBB4; EGFR; CALM2; AR |
| C97 | Linarin | 10 | SOD2; PAH; NOS2; MET; IMPA2; GSK3B; GLO1; ESR2; ANG; ALDH2 |
| C98 | Linoleyl acetate | 2 | PTGS2; PTGS1 |
| C99 | liquiritin | 19 | TTR; TGFBR2; SOD2; SOD1; SHBG; PTGS2; PAH; NR3C2; KDR; IMPA2; GSR; GLO1; GBA; ESR1; DPP4; CALM2; ANG; ALDH2; AKR1C3 |
| C100 | Longikaurin A | 4 | GABRA6; GABRA3; GABRA2; CHRM2 |
| C101 | Lupiwighteone | 15 | PTGS2; PLA2G10; NOS2; MET; MAPK14; HSD11B1; GSK3B; FGFR1; ESR2; ESR1; DUSP6; DPP4; CALM2; AR; AKR1C3; AKR1C1 |
| C102 | luteolin | 27 | VEGFA; TP53; TNF; PTGS2; PTGS1; PRKACA; PAH; NR3C2; NOS3; MIF; MET; MCL1; MAPK1; IL6; IL4; IL10; IFNG; GSR; ESR2; ESR1; EGFR; DUSP6; DPP4; BCL2L1; AR; APP; ANG; AKT1 |
| C103 | Glycyrrhiza flavonol A | 8 | PTGS2; NOS2; GSK3B; ESR2; ESR1; DPP4; CALM2; AR; ACHE |
| C104 | Mairin | 12 | WAS; RORA; NR3C2; NOS3; MTHFD1; MAPK8; MAPK14; KDR; GSK3B; FABP3; ALB; AKR1C1; ADH1B |
| C105 | Medicarpin | 22 | SLC6A4; SLC6A3; PTGS2; PTGS1; PRKACA; OPRM1; OPRD1; NOS3; NOS2; HTR2A; HSD11B1; GSR; GSK3B; ESR2; ESR1; DUSP6; DRD1; DPP4; CHRM2; CALM2; AR; ADRB2; ADRA1A |
| C106 | naringenin | 24 | SOD1; SHBG; PTGS2; PTGS1; PRKACA; PLB1; NOS3; MTHFD1; MIF; MAPK3; MAPK14; MAPK1; KDR; GSR; ESR2; ESR1; DUSP6; CAT; BCL2; AR; ANG; AKT1; AKR1C1; ADIPOQ; ABAT |
| C107 | Odoratin | 16 | SHBG; PTGS2; PTGS1; PAH; NR3C2; NR1H2; NOS2; MAPK14; LTA4H; HSP90AA1; GSK3B; ESR2; ESR1; DPP4; CALM2; AR; AKR1C1 |
| C108 | paeoniflorgenone | 22 | WAS; SOD2; SHBG; RORA; PAH; NR3C2; MIF; MET; KDR; IGF1R; HSP90AA1; HSD11B1; GSK3B; GABRA1; FGFR2; ESR2; ERBB4; EGFR; CYP2C9; CD1A; AR; ALB; AKR1C1 |
| C109 | paeoniflorin | 28 | WAS; VDR; TNF; SOD2; RORA; REN; NR3C2; MME; MIF; LBP; KDR; IL6; IGF1R; HSP90AA1; HSD11B1; GSK3B; ESR2; ERBB4; EGFR; DPP4; CYP2C9; CTSF; CD1A; AR; ALDH2; ALB; AKT1; AKR1C3; AKR1C1 |
| C110 | petunidin | 6 | PTGS2; PTGS1; NOS2; MAPK14; GSK3B; ESR2 |
| C111 | Phaseol | 19 | TTR; SHBG; PTGS2; PRKACA; PLA2G10; MAPK14; MAPK1; KDR; IGF1R; HSD11B1; GSK3B; FGFR2; ESR2; ESR1; ERBB4; DPP4; CYP2C9; CALM2; AR; ALB; |
| C112 | Phaseolinisoflavan | 9 | PTGS2; NOS2; MAPK14; GSK3B; ESR2; ESR1; CALM2; AR; ADRB2; ACHE |
| C113 | quercetin | 39 | VEGFA; TP53; TNF; PTGS2; PTGS1; PRKACA; PAH; NR3C2; NOS3; MIF; MET; MCL1; MAPK1; IL6; IL4; IL10; IFNG; GSR; ESR2; ESR1; EGFR; DUSP6; DPP4; BCL2L1; AR; APP; ANG; AKT1 |
| C114 | Quercetin der. | 10 | PTGS2; PTGS1; NOS2; MAPK14; GSK3B; ESR2; ESR1; DPP4; CALM2; AR |
| C115 | shinpterocarpin | 27 | SHBG; RORA; PTGS2; PTGS1; PRKACA; OPRM1; OPRD1; NR3C2; NOS2; MIF; MET; MAPK8; MAPK14; MAPK1; HTR3A; HSP90AA1; HSD11B1; GSR; GSK3B; ESR2; ESR1; DUSP6; CALM2; AR; ALB; AKR1C1; ADRB2 |
| C116 | Sigmoidin-B | 16 | SOD2; SHBG; REN; PTGS2; PLA2G2A; NR3C2; MET; KDR; HSD11B1; GSR; GSK3B; FGFR1; ESR2; ESR1; CALM2; AKR1C1 |
| C117 | Saikosaponin D | 3 | DNMT1; ADORA2A; ADORA1 |
| C118 | Stigmasterol | 26 | SLC6A3; SLC6A2; SHBG; RORA; PTGS2; PTGS1; PRKACA; NR3C2; MET; MAOB; MAOA; LTA4H; HTR2A; HSD11B1; GABRA3; GABRA1; ESR1; ERBB4; DPP4; CHRM2; CALM2; AKR1C1; ADRB2; ADRB1; ADRA2A; ADRA1A |
| C119 | trametenolic acid | 17 | WAS; SHBG; RORA; REN; NR3C2; NOS3; MIF; MAPK14; MAOB; HSP90AA1; HSD11B1; GSK3B; ESR1; CALM2; ALB; AKR1C1; ADH1B |
| C120 | Vestitol | 20 | SLC6A4; SLC6A3; PTGS2; PTGS1; PRKACA; NOS2; MAPK14; HTR2A; HSP90AA1; HSD11B1; GSK3B; FGFR1; ESR2; ESR1; DPP4; CALM2; AR; ALB; ADRB2; ADRA1A |
| C121 | Xambioona | 18 | WAS; PTGS2; NR3C2; NOS2; MAPK8; MAPK14; MAOB; KDR; HSP90AA1; HSD11B1; ESR2; ESR1; EGFR; DPP4; CALM2; AR; ALB; AKR1C1 |
