# Supplementary material for: An Integrated Pharmacology-Based Analysis for Antidepressant Mechanism of Chinese Herbal Formula Xiao-Yao-San
Source: Front Pharmacol. 2020 Mar 18;11:284. doi: 10.3389/fphar.2020.00284 (PMC7094752; doi:10.3389/fphar.2020.00284)
Supplement: Table S5 — Compounds acting on hub targets. [file Table_5.DOCX]

**Table S5. Compounds acting on hub targets**

| **Hub target** | **Compoud** |
| --- | --- |
| AKT1 | luteolin |
| AKT1 | naringenin |
| AKT1 | paeoniflorin |
| AKT1 | quercetin |
| AKT1 | gadelaidic acid |
| AKT1 | Glyasperin C |
| AKT1 | kaempferol |
| TP53 | acacetin |
| TP53 | aloe-emodin |
| TP53 | luteolin |
| TP53 | quercetin |
| VEGFA | luteolin |
| VEGFA | quercetin |
